# A 66‐Nuclear All‐Alkynyl Protected Peanut‐Shaped Silver(I)/Copper(I) Heterometallic Nanocluster: Intermediate in Copper‐Catalyzed Alkyne‐Azide Cycloaddition

**DOI:** 10.1002/advs.202400377

**Published:** 2024-04-01

**Authors:** Jin‐Ping Gao, Fu‐Qiang Zhang, Xian‐Ming Zhang

**Affiliations:** ^1^ School of Chemistry & Material Science Shanxi Normal University Taiyuan 030006 P. R. China; ^2^ College of Chemistry Taiyuan University of Technology Taiyuan 030024 P. R. China

**Keywords:** 1,3‐dipole cycloadditions, CuAAC reaction, Heterometallic nanoclusters, Intermediate

## Abstract

Ligand‐protected heterometallic nanoclusters in contrast to homo‐metal counterparts show more broad applications due to the synergistic effect of hetero‐metals but their controllable syntheses remain a challenge. Among heterometallic nanoclusters, monovalent Ag‐Cu compounds are rarely explored due to much difference of Ag(I) and Cu(I) such as atom radius, coordination habits, and redox potential. Encouraged by copper‐catalyzed alkyne‐azide cycloaddition (CuAAC) reaction, comproportionation reaction of Cu(II)X_2_ and Cu(0) in the presence of (PhC≡CAg)_n_ complex and molybdate generated a core‐shell peanut‐shaped 66‐nuclear Ag(I)‐Cu(I) heterometallic nanocluster, [(Mo_4_O_16_)_2_@Cu_12_Ag_54_(PhC≡C)_50_] (referred to as **Ag_54_Cu_12_**). The structure and composition of Ag‐Cu heterometallic nanocluster are fully characterized. X‐ray single crystal diffraction reveals that **Ag_54_Cu_12_** has a peanut‐shaped silver(I)/copper(I) heterometallic nanocage protected by fifty phenylacetylene ligands in *µ*_3_–modes and encapsulated two mutually twisted tetramolybdates. Heterometallic nanocage contains a 54‐Ag‐atom outer ellipsoid silver cage decorated by 12 copper inside wall. Nanosized **Ag_54_Cu_12_** is a *n*‐type narrow‐band‐gap semiconductor with a good photocurrent response. Preliminary experiments demonstrates that **Ag_54_Cu_12_** itself and activated carbon supported **Ag_54_Cu_12_/C** are effective catalysts for 1,3‐dipole cycloaddition between alkynes and azides at ambient conditions. The work provides not only a new synthetic route toward Ag(I)‐Cu(I) nanoclusters but also an important heterometallic intermediate in CuAAC catalytic reaction.

## Graphical abstract

A 66‐nuclear all‐alkynyl protected (Mo_4_O_16_)_2_@Cu_12_Ag_54_ (**Ag_54_Cu_12_**) nanocluster is isolated by copper‐catalyzed alkyne‐azide cycloaddition (CuAAC) reaction by using silver‐phenylacetylene precursor during CuAAC reaction without azides. **Ag_54_Cu_12_** has a peanut‐shaped Ag(I)/Cu(I) nanocage protected by fifty PhC≡C^−^ ligands in *µ*_3_–modes and encapsulates two mutually twisted Mo_4_O_16_^8−^. **Ag_54_Cu_12_** itself and **Ag_54_Cu_12_/C** are effective catalysts for 1,3‐dipole cycloaddition reaction at ambient conditions.

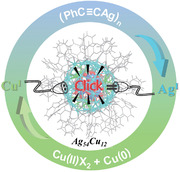

## 1 Introduction

High‐nuclearity ligand‐protected heterometallic nanoclusters are more attractive than homo‐metal nanoclusters for potential applications such as catalysis, photoluminescence, electrochemistry, and other fields, which is due to the synergistic effect of hetero‐metal nanoclusters on physicochemical properties.^[^ ^1^ ^]^ The key to tuning physicochemical behavior of heterometallic clusters is the preparation of nanoclusters with controllable composition/doping sites.^[^ ^2^ ^]^ Generally, heteroatoms that can be dope into silver nanoclusters are noble metals with similar radius in the periodic table.^[^ ^3^ ^]^ Conventional doping methods are based on co‐reduction, metal exchange, metal deposition of atoms/ions [(also known as anti‐galvanic reaction (AGR)], or a combination of these strategies.^[^ ^4^ ^]^ These strategies are less effective in controlling reduction of double metal salts and formation of heteroatomic nanoclusters. A search reveals that the availability of heteronuclear Ag(I)‐Cu(I) alkynyl clusters via Cu doping are rare.^[^ ^5^ ^]^ As far as dopants of Cu element are concerned, the standard potential of Cu^2+^/Cu^+^ couple is as low as 159 mV, which indicates that the +1 oxidation state of copper is susceptible to be oxidized to much more stable +2 state. Furthermore, the aggregative nature of Cu(I) alkynyl causes a larger challenge in isolation of discrete Ag(I)‐Cu(I) alkynyl nanoclusters. To solve this puzzle, the comproportionation reaction based copper‐catalyzed alkyne‐azide cycloaddition (CuAAC) reaction could provide a route toward stable homogeneous high‐nuclearity monovalent copper alkynyl clusters.^[^ ^6^ ^]^ The origin of Cu(I) intermediate is generated by Cu(II) with various Cu(0) sources such as wire, turnings, powder and nanoparticles in CuAAC reaction. This reaction could overcome potential difference to produce monovalent copper,^[^ ^7^ ^]^ which in combination with a variety of alkynyl precursors give metal nanoclusters with tunable composition, structure, and properties.^[^ ^8^ ^]^ In spite of success in homogeneous copper, the implementation of the experiments remains a challenge in heterogeneous Cu/Ag nanoclusters(.

On the other hand, Cu(I) alkynyl complexes have been extensively studied as key intermediates in copper‐catalyzed transformations of alkynes as well as in click chemistry synthesis of 1,2,3‐triazole.^[^ ^9^ ^]^ The widely utilized CuAAC involves generation of Cu^I^‐alkynyl species of various nuclearities.^[^ ^10^ ^]^ Reported examples include Cu_33_ and Cu_62_,^[^ ^11^ ^]^ and Cu_20_^[^ ^12^ ^]^ nanocluster.^[^ ^13^ ^]^ Apart from these homogeneous intermediates, there are only sporadic reports on heterometallic intermediates involving the click reaction. However, understanding of interaction of Cu(I) and hetero‐metals in heterogeneous CuAAC catalytic systems is not well clear. Recently, Zhu et al captured three crucial Au_4_Cu_4_−π‐alkyne intermediates and discussed an abnormal mechanism in CuAAC reaction, which is different from comproportionation reaction to dehydrogenate.^[^ ^14^ ^]^ Therefore, the revealing of Cu(I) and Ag(I) interaction in heterometallic Cu/Ag clusters is of significance in click chemistry of CuAAC reaction. It is still an open question whether heterometallic intermediates can be achieved by comproportionation reaction and alkyne precursor. Inspired by the widely used methodology for Cu‐ethyne nanoclusters, we would like to explore the similar story in Ag‐ethyne cousins.

**Scheme 1:**
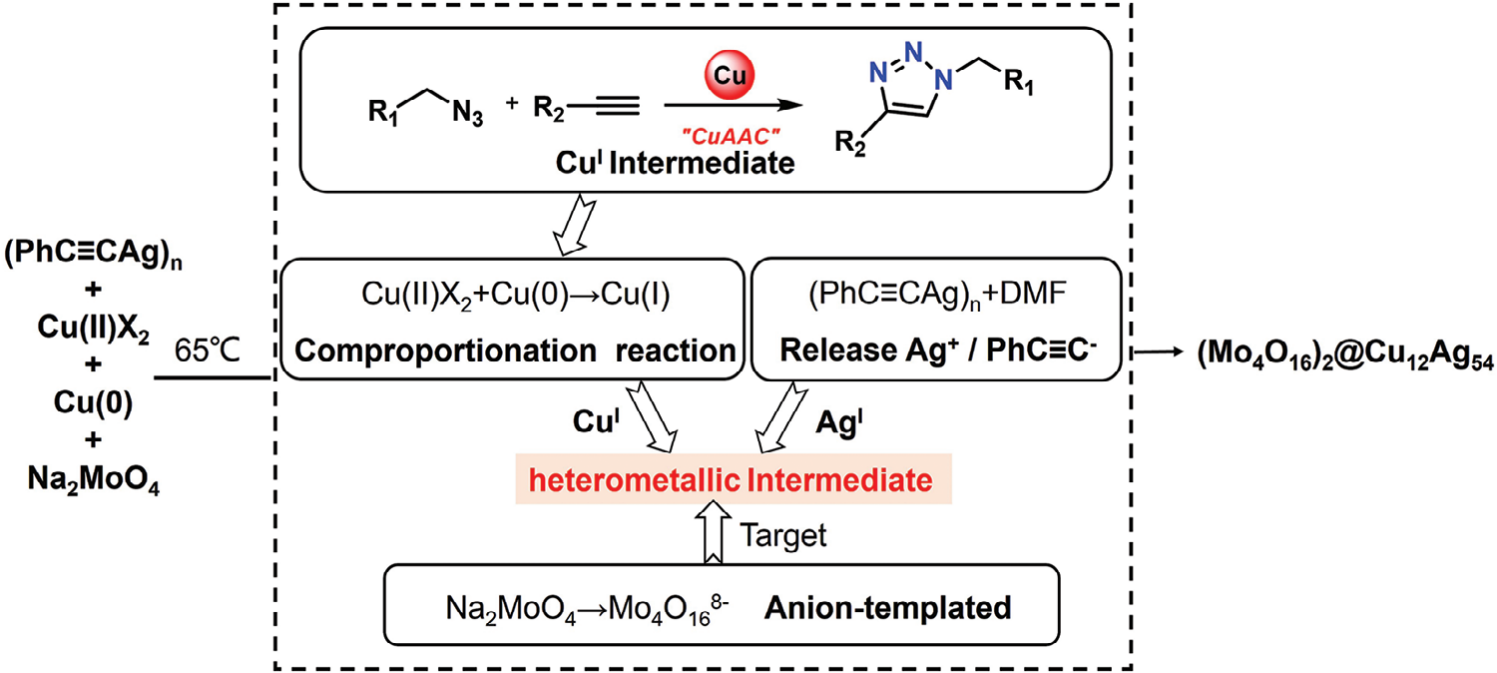
The synthesis route of **Ag_54_Cu_12_** nanocluster.

In order to generate new heterometallic nanoclusters and intermediates in CuAAC reaction, a series of Ag‐Cu‐alkyne products in the system need to be obtained by alkyne ligands. Our strategy is to creatively use silver‐phenylacetylene precursor instead of alkyne or copper‐alkyne ligand during CuAAC reaction without azides. Silver‐phenylacetylene precursor provides alkyne source for CuAAC intermediate because alkynyl as a π‐acid ligand can bind to d^10^ ions of Au, Ag, Cu via σ‐π modes and form various metal‐carbon interactions.^[^ ^15^ ^]^ As such, the pronounced interactions of alkynyl in a variety of coordination modes may affect the physicochemical properties of metal clusters due to metal atom kernel and metal‐alkyne interactions.^[^ ^16^ ^]^ This allows the atomic‐level understanding of structure‐property correlations, which in turn favours the targeted preparation of metal nanoclusters.^[^ ^17^ ^]^ In addition, the slow release of Ag^+^ from silver‐phenylacetylene in a weakly reducing DMF can bind Cu^+^ in situ.^[^ ^18^ ^]^ As in **Scheme** **1**, we envisage that the polymolydate anion template will target Ag and Cu metal centres to aggregate in a core‐shell structure, resulting in the formation of Ag‐Cu clusters.

Herein, we present a 66‐nuclear all‐alkynyl protected peanut‐shaped silver(I)/copper(I) heterometallic nanocluster, [(Mo_4_O_16_)_2_@Cu_12_Ag_54_(PhC≡C)_50_] (**Ag_54_Cu_12_)**, which was synthesized by using sliver‐alkyne complex and protective alkyne ligands in the presence of molybdate. The two tetramolybdates [Mo_4_O_16_]^8−^ mutually twisted ca. 24° were encapsulated by Cu_12_Ag_54_ cage to form a core‐shell peanut‐shaped (Mo_4_O_16_)_2_@Cu_12_Ag_54_ nanocluster. The surface PhC≡C^−^ ligands are ligated Ag and Cu in five types coordination modes to stabilize the nanocluster. The structure and composition of **Ag_54_Cu_12_** were determined by high‐resolution electrospray ionization mass spectrometry (ESI‐TOF‐MS), X‐ray diffraction (XRD), energy‐dispersive X‐ray spectroscopy (EDS), Fourier‐transform infrared (FT‐IR) spectroscopy, and X‐ray photoelectron spectroscopy (XPS). Furthermore, a solid‐state UV–vis absorption spectrum of **Ag_54_Cu_12_** showed a narrow‐bandgap semiconductor with a good photocurrent response. **Ag_54_Cu_12_** itself and **Ag_54_Cu_12_** loaded with actived carbon as catalysts were applied in [3+2] cycloaddition reactions between alkynes and azides at ambient conditions.

## 2 Results and Discussion

**Figure 1:**
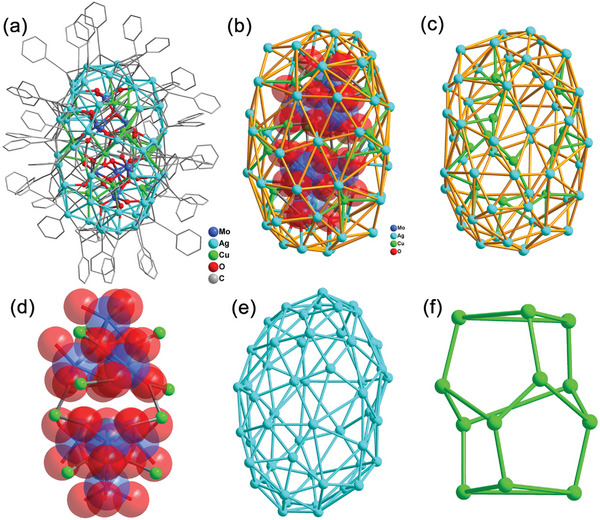
a) Full structure of **Ag_54_Cu_12_** nanocluster. b) The core‐shell peanut shaped of **Ag_54_Cu_12_**. c) The Cu_12_Ag_54_ ellipsoid cage as peanut shell. d) The two tetrabolydates serve as seeds for the nanocluster. e) Ag_54_ shell. f) Cu_12_ motif.

Single X‐ray diffraction analysis indicated **Ag_54_Cu_12_** nanocluster crystalized in triclinic *P*1 space group.^[^ ^19^ ^]^ The asymmetry unit contains a whole **Ag_54_Cu_12_** cluster (**Figure** **1**a). The shape of the 66‐nucleus cluster resembles a peanut with Cu_12_Ag_54_ ellipsoidal cage as peanut shell and two Mo_4_O_16_^8−^ groups as peanut seeds (Figure 1b). Notably, the nuclearity of **Ag_54_Cu_12_** is comparable with those of the previously reported monovalent Ag‐Cu heterometallic nanocluster, namely [Ag_74‐x_Cu_x_O_12_(PhC≡C)_50_]_27_^[^ ^18a^ ^]^ and [Ag_40.13_Cu_13.87_S_19_(tBuS)_20_(tBuSO_3_)_12_].^[^ ^20^ ^]^ The overall structure contains 54 Ag atoms, 12 Cu atoms, 50 PhC≡C^−^ ligands, and two Mo_4_O_16_ units (Figure 1a). The Cu_12_Ag_54_ cage consists of an Ag shell on the periphery and Cu embedded in the inner shell, which generates Ag‐Cu heterometallic interactions (Figure 1c,d). The shell of peanut‐shaped Cu_12_Ag_54_ consists of silver triangles and tetragons with short Ag···Ag interactions of 2.84–3.41 Å (Figure 1e), whose surface is covered by 50 PhC≡C^−^ groups in *µ*_3_–*η* ^1^:*η* ^1^:*η* ^1^, *µ*_3_–*η* ^1^:*η* ^1^:*η* ^2^, *µ*_3_–*η* ^1^:*η* ^2^:*η* ^2^, and *µ*_3_–*η* ^2^:*η* ^2^:*η* ^2^ coordination modes with Ag‐C distances of 2.01(2)−2.704(16) Å and Cu‐C distances of 1.815(18)–1.888(13) Å. Inner wall twelve Cu atoms coordinate to carbon atoms of PhC≡C^−^ and oxygen atoms of Mo_4_O_16_^8−^ group, which are interacted with Ag atoms of shell with short Cu···Ag distances of 2.734(3)−3.056(3). Considering the arrangement of copper atoms, twelve Cu atoms form a Cu_12_ motif comprised of pentagons and triangles (Figure 1f). The 3D packing modes of **Ag_54_Cu_12_** nanoclusters in *b* and *c* directions are shown in Figure S1 (Supporting Information).

The peanut kernel consists of two independent Mo_4_O_16_^8−^ tetramolybdates as seeds (Figure S3a,b, Supporting Information), which show similar cubane‐like structures with *T*_d_ symmetry, with Mo─O bond distances ranging from 1.705(10) to 2.288(19) Å.^[^ ^21^ ^]^ The bond valence for Mo ions by valence sum (BVS) calculations (Table S2, Supporting Information) demonstrated +6. The [Mo_4_O_16_]^8−^ anion was self‐assembled in situ from molybdate during the solvothermal process. Surprisingly, in contrast to the orientation of Mo_4_O_16_^8−^ groups in our recently reported [Cu_3_Mo_8_O_32_]^10−^,^[^ ^22^ ^]^ the two Mo_4_O_16_^8−^ groups in **Ag_54_Cu_12_** are not overlapped but staggered by ≈24 degrees (Figure S3c, Supporting Information).

Compared to reported metal nanoclusters encapsulating multiple polyoxometallates (POMs), the twisted configuration of two unconnected [Mo_4_O_16_]^8‐^ units is unique (reports listing these high nuclear clusters are in Table S3, Supporting Information). The unconnected arrangement of two POMs allows more oxygen sites to be exposed, which is beneficial for enhancing the template effect. Careful check revealed that two [Mo_4_O_16_]^8−^ units are bonded to 12 Cu atoms through 6 *µ*_2_‐O and 24 terminal oxygen atoms via weak Cu···O bonds, and to 34 Ag atoms via 20 terminal oxygen atoms. The 20 Ag atoms uncoordinated to POMs were found to be embedded into Ag_54_ shell by Ag─Cu, Ag─C, and Ag─Ag interactions. The Cu_12_Ag_54_ shell was further consolidated by all alkyne ligands.

**Figure 2:**
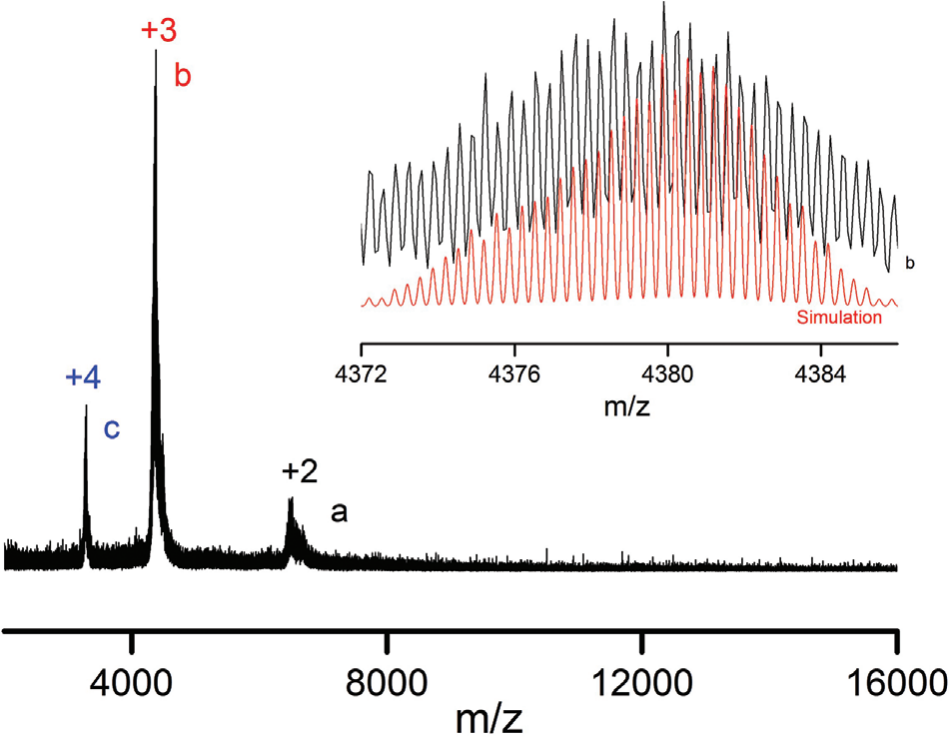
ESI‐TOF‐MS spectra of **Ag_54_Cu_12_**.

To investigate the solution behavior and composition, **Ag_54_Cu_12_** cluster was monitored utilizing electrospray ionization‐time of flight‐mass spectrometry (ESI‐TOF‐MS) in the mass‐to‐charge ratio (m/z) range of 1000–20000. As shown in **Figure** **2**, **Ag_54_Cu_12_** was dissolved in a mixed solvent of methanol and dichloromethane. In positive ion mode, peaks with two valence states were observed in the m/z ranges of 3400–3500 and 4200–4400, respectively. The main peak b3 at the highest abundance m/z = 4379.85 can be attributed to [(Mo_4_O_16_)_2_@Cu_12_Ag_54_(PhC≡C)_50_(CH_3_OH)(CH_3_OH_2_)_3_]^3+^ (*cal*. 4379.91), which was considered to be the **Ag_54_Cu_12_** molecular ion peak with three protonated MeOH molecules.

The composition of hetero‐metal cluster was supported by PXRD and FI‐IR spectra (Figures S4 and S5, Supporting Information, Experimental Section). XPS spectra shown the presence of Ag, Cu, Mo, C, and O elements, which was consistent with the results of EDS‐mapping (Figures S6 and S7a, Supporting Information). High‐resolution spectra clearly illustrated the valences of metal ions of **Ag_54_Cu_12_**. As shown in Figure S7b (Supporting Information), XPS data of the Ag 3d_5/2_ and Ag 3d_3/2_ binding energies are 368.26 and 374.24 eV, respectively, confirming that Ag atoms in the cluster are positively charged.^[^ ^23^ ^]^ The Mo binding energy at 232.9 and 235.2 eV in Figure S7c (Supporting Information) can be attributed to Mo^6+^ 3d_5/2_ and 3d_3/2_ spin‐splitting slits.^[^ ^24^ ^]^ Figure S7d (Supporting Information) shows XPS peaks of monovalent Cu(I). The Cu LMM Auger chemical shift also showed monovalent Cu^I^ state (Figure S9, Supporting Information).

**Figure 3:**
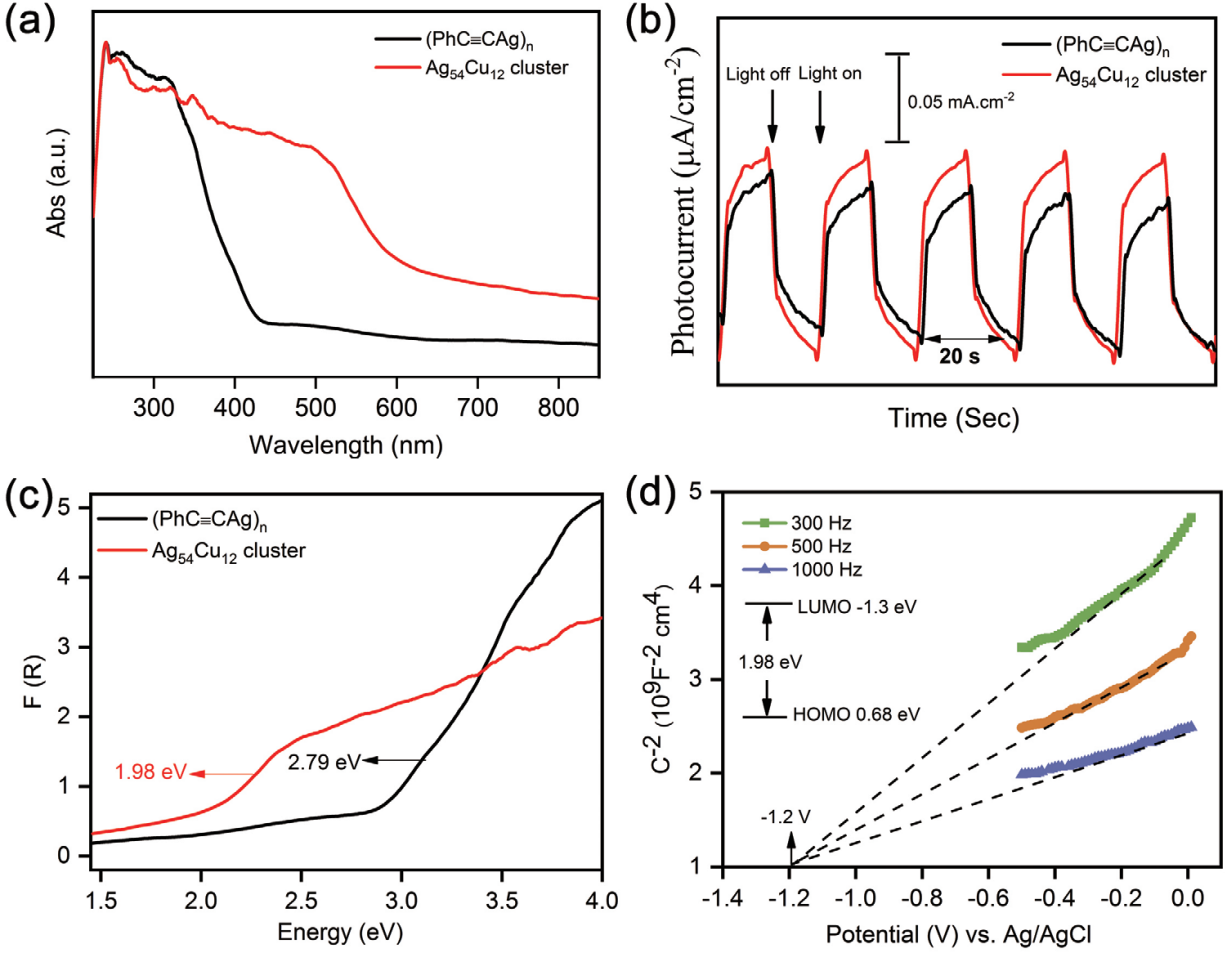
a) Solid‐state UV–vis absorption spectra and b)photocurrent responses under UV irradiation of (PhC≡CAg)_n_ and **Ag_54_Cu_12_**. c) Kubelka‐Munk function versus energy (eV) and Tauc plots of (PhC≡CAg)_n_ and **Ag_54_Cu_12_**. d) Mott‐Schottky plot of **Ag_54_Cu_12_** in a 0.2 m Na_2_SO_4_ aqueous solution.

The solid‐state UV absorption spectra of **Ag_54_Cu_12_** show a broadband absorption in the wavelength range 300–400 nm, which is attributed to the π→π* transition due to appearance of similar band in (PhC≡CAg)_n_ precursor. The optical bandgap of **Ag_54_Cu_12_** was determined by Tauc equation to be 1.98 eV, which is narrower than that of 2.79 eV in precursor (PhC≡CAg)_n_ (**Figure** **3**c). This suggests that **Ag_54_Cu_12_** has potential as a narrow‐band‐gap semiconductor.^[^ ^18^, ^25^ ^]^ The photoelectrochemical behavior of **Ag_54_Cu_12_** was tested in a typical three‐electrode system in a 0.2 m Na_2_SO_4_ aqueous solution. Compared with (PhC≡CAg)_n_, an obvious photocurrent response was detected upon on‐off cycling irradiation, indicating a better electron and hole separation efficiency of **Ag_54_Cu_12_**. The photocurrent density could reach up to 0.11 *µA* cm^−2^, which remained nearly constant with increased test times, indicating high photophysical stability of **Ag_54_Cu_12_**. Considering the board absorption semiconductor property of **Ag_54_Cu_12_**, Mott‐Schottky (M‐S) measurements were performed at frequencies of 300, 500 and 1000 Hz in darkness (Figure 3d). The positive slope of the C^−2^‐E plot confirms that **Ag_54_Cu_12_** is an *n*‐type semiconductor.^[^ ^26^ ^]^ The flat band potential (EFB) was determined by the intersection to be ≈−1.3 V versus Ag/AgCl, corresponding to a potential of −0.68 V versus NHE. It is expected that Based on the previous reports, the conduction band edge of semiconductor should be ≈0.10 V more negative than the EFB. Therefore, it can be estimated that the conduction band (LUMO) of **Ag_54_Cu_12_** is approximately −1.2 V versus NHE. The valence band (HOMO) of **Ag_54_Cu_12_** is estimated to be 0.68 V versus NHE.

**Figure 4:**
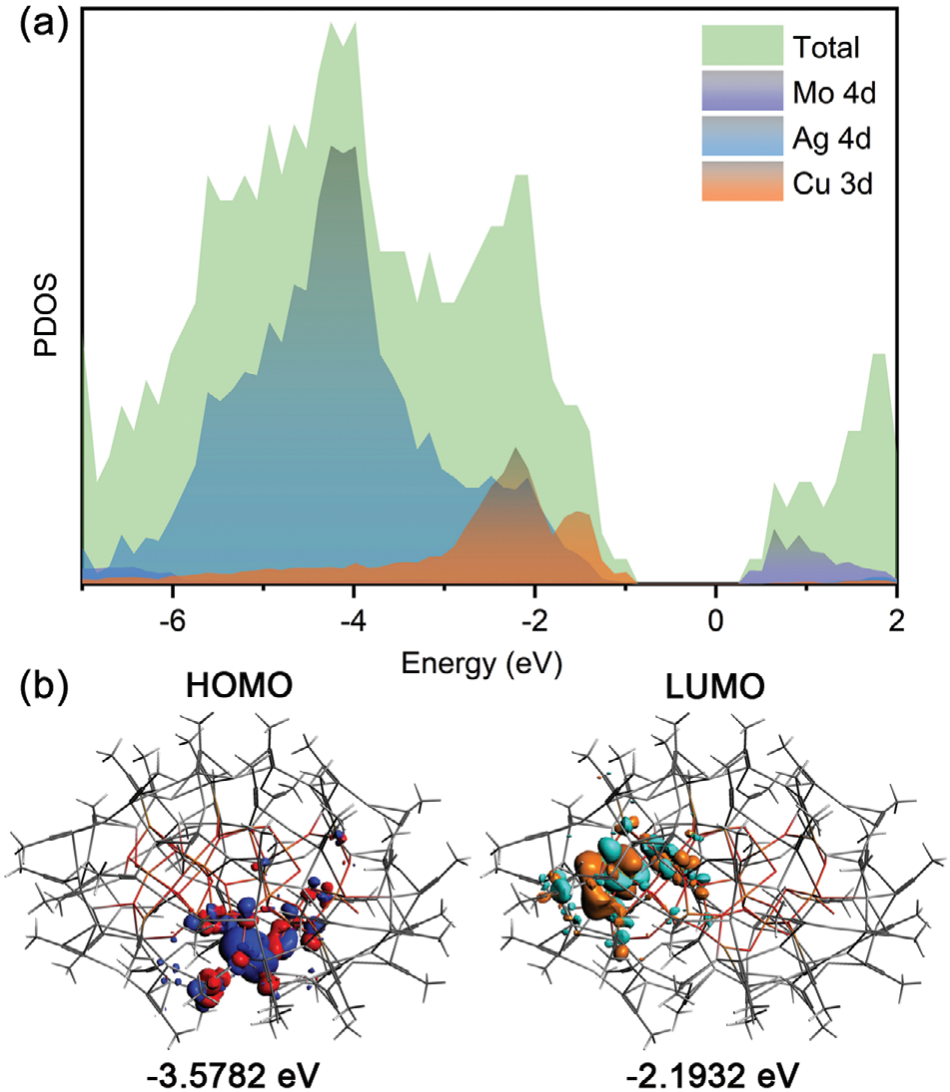
a) Total and partial DOS of **Ag_54_Cu_12_** cluster. b) Frontier molecular orbitals: HOMO and LUMO.

To understand the role of electron orbitals around the Fermi level, the total and partial densities of states (DOSs) of **Ag_54_Cu_12_** were calculated. As is shown **Figure** **4**a, the top of the valence band is mainly composed of metal Ag 4d, Cu 3d, and a few Mo 4d states. The bottom of the conduction band is dominated by metal Mo 4d, a few Ag 4d and Cu 3d, proving that the optical properties of **Ag_54_Cu_12_** are determined by Ag, Cu and [Mo_4_O_16_]^8−^ anion template. The HOMO‐LUMO bandgap difference is calculated to be 1.383 eV, in well agreement the experimental value. The lowest empty orbital of **Ag_54_Cu_12_** is primarily located in the Mo 4d orbital on [Mo_4_O_16_]^8−^, while the highest occupied orbital is mainly concentrated in the Mo 4d and C 2p of PhC≡C^−^ (Figure 4b). This indicates that [Mo_4_O_16_]^8−^ anion has a significant effect on the optical properties of **Ag_54_Cu_12_**. Several high lying occupied orbitals, such as HOMO‐1, HOMO‐2, HOMO‐3, and HOMO‐4, are almost entirely contributed by orbitals of peripheral ligand C≡C bonds (Figure S9, Supporting Information). According to Frontier molecular orbitals theory, LUMO is logically considered electrophilic, which may partly explain the fact that the outer O atoms of the non‐coordinated/exposed portion of Mo_4_O_16_^8−^ unit are less likely to further coordinate with the Ag atoms.

Copper‐catalyzed [3+2] Huisgen cycloadditions of terminal alkynes and organic azides (CuAAC) were the cornerstone of Meldal and Sharpless research, most of which involved homometallic Cu‐based materials.^[^ ^27^, ^28^ ^]^ To investigate and develop heterometallic 1,3‐dipole cycloaddition reaction catalysis, **Ag_54_Cu_12_** was implemented by using phenylacetylene and benzyl azide at 40 °C as the model reaction. This reaction exhibits completely regioselectivity and is a powerful method for the rapid assembly of 1,4‐disubstituted‐1,2,3‐triazoles. The solution of **Ag_54_Cu_12_** cluster was gradually added to a suspension of activated carbon/titanium dioxide in ethanol, and resulted mixture was stirred and centrifuged to give C/TiO_2_ supported nanocluster catalysts. Transmission electron microscopy (TEM) illustrated that all particles of as‐synthesized products were less than 2 nm in size, indicating good dispersion of the clusters loaded on the activated carbon (Figure S11, Supporting Information).

First, the influence of different solvents on the reaction was investigated. A relatively higher yield was realized in CH_3_CN (99%), compared to CH_3_OH solvent (**Table** **1**, entries 1–5). In addition, the effect of supports and loading capacity was also investigated under the same reaction conditions, and a high isolated yield of 99% was realized (Table 1, entry 1–2 and 4–5). The unsupported **Ag_54_Cu_12_** NCs gave a lower isolated yield of 88% (Table 1, entry 6). Importantly, the control experiments showed that **Ag_54_Cu_12_** significantly contributes to the cycloaddition process (Table 1, entries 7–11).

**Table 1:** Cycloaddition of phenylacetylene and benzyl azide.

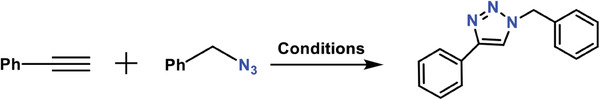						
Entry^a)^	Catalyst	Cu Loading [mmol, %]^b)^	Solvent	Time	Yield [%]^c)^	TOF
1	**Ag_54_Cu_12_/C**	0.23	CH_3_CN	12 h	80%	28.98
2	**Ag_54_Cu_12_/C**	0.46	CH_3_CN	12 h	99%	17.93
3	**Ag_54_Cu_12_/C**	0.23	CH_3_OH	12 h	45%	16.30
4	**Ag_54_Cu_12_/TiO_2_**	0.23	CH_3_CN	12 h	13%	4.71
5	**Ag_54_Cu_12_/TiO_2_**	0.46	CH_3_CN	12 h	70%	12.68
6	**Ag_54_Cu_12_**	2.3	CH_3_CN	12 h	88%	3.19
7	C	—	CH_3_CN	24 h	0	—
8	TiO_2_	—	CH_3_CN	24 h	0	—
9	AgSO_3_CF_3_	—	CH_3_CN	24 h	0	—
10	Na_2_MoO_4_	—	CH_3_CN	24 h	0	—
11	None	—	CH_3_CN	24 h	0	—

^a)^ Reaction conditions: phenylacetylene (0.22 mmol), benzyl azide (0.2 mmol), acetonitrile (1 mL), 40 °C.

^b)^ Catalyst loading defined as mmol% Cu‐based **Ag_54_Cu_12_** cluster.

^c)^ Isolated yield.

Based on optimized standard conditions, the generality of cycloaddition was explored for various aryl‐terminated acetylenes and the corresponding results were summarized in **Table** **2**. Good yields (90%–94%) were obtained for substituted aryl alkynes with both electron‐donating (–OCH_3_ and –CH_3_) and electron‐withdrawing (–F, –Cl, and –NO_2_) substituents (Table 2, entries 1–6). These results demonstrated the important interaction between **Ag_54_Cu_12_** and activated carbon substrate.

**Table 2:** Scope of benzyl azide with different alkynes.

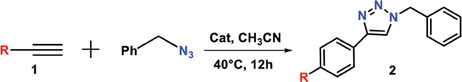 ^a)^,^b)^
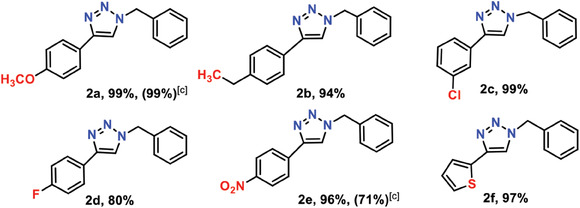 ^c)^

^a)^ Reaction conditions: phenylacetylene (0.22 mmol), benzyl azide (0.2 mmol), acetonitrile (1 mL), **Ag_54_Cu_12_/C** (Cu loading 0.46% mmol), 40 °C.

^b)^ Isolated yield.

^c)^ Reaction conditions: phenylacetylene (0.22 mmol), benzyl azide (0.2 mmol), acetonitrile (1 mL), **Ag_54_Cu_12_** (Cu loading 2.3% mmol), 40 °C.

Interestingly, some 1,2,3‐triazole products were crystallized from reaction environment (Tables 1, entry 6 and 2, entries 7–8). The single crystal data of products were collected and solved (Figure S12a and Tables S5 and S6, Supporting Information), directly confirming their corresponding identity. Both **Ag_54_Cu_12_** and **Ag_54_Cu_12_/C** as catalysts exhibited excellent cycle stability after six experiments (Figure S12b, Supporting Information). These results demonstrated that **Ag_54_Cu_12_** NCs could be a high‐ performance molecular catalysts for CuAAC.

## 3 Conclusion

In conclusion, we have isolated an all‐alkyne protected silver(I)/copper(I) heterometallic nanocluster, [(Mo_4_O_16_)_2_@Cu_12_Ag_54_(PhC≡C)_50_] (**Ag_54_Cu_12_**), which was synthesized by the reduction of Cu (II) salt and copper powder in presence with (PhC≡CAg)_n_ and Na_2_MoO_4_ under solvothermal method. Two mutually twisted [Mo_4_O_16_]^8−^ anions were encapsulated by Cu_12_Ag_54_ cage to form a core‐shell peanut‐shaped (Mo_4_O_16_)_2_@Cu_12_Ag_54_ nanocluster. The surface PhC≡C^−^ ligands are ligated to Ag and Cu in σ‐π modes to stabilize the nanocluster. XRD, XPS, and ESI‐TOF‐MS certify the structure and composition of **Ag_54_Cu_12_**. The *n*‐type narrow‐band‐gap material was confirmed by solid state UV–vis spectra and DFT calculation. Furthermore, **Ag_54_Cu_12_** itself and **Ag_54_Cu_12_/C** are good catalysts for 1,3‐dipole cycloaddition between alkynes and azides at ambient conditions. Different from conventional methods for heterometallic clusters, the comproportionation reaction in the work could provide an effective method in controlling reduction of double metal salts and formation of heteroatomic high‐nuclearity monovalent copper alkynyl clusters.

## Conflict of Interest

The authors declare no conflict of interest.

## Supporting information

Supporting Information

Supporting Information

## Data Availability

The data that support the findings of this study are available from the corresponding author upon reasonable request.
