# Supplementary material for: A 66‐Nuclear All‐Alkynyl Protected Peanut‐Shaped Silver(I)/Copper(I) Heterometallic Nanocluster: Intermediate in Copper‐Catalyzed Alkyne‐Azide Cycloaddition
Source: Adv Sci (Weinh). 2024 Apr 1;11(22):2400377. doi: 10.1002/advs.202400377 (PMC11165478; doi:10.1002/advs.202400377)
Supplement: Supplementary file 1 — Supporting Information [file ADVS-11-2400377-s001.pdf]

ADVANCED SCIENCE
Open Access

Supporting Information

for *Adv. Sci.*, DOI 10.1002/advs.202400377

# A 66-Nuclear All-Alkynyl Protected Peanut-Shaped Silver(I)/Copper(I) Heterometallic Nanocluster: Intermediate in Copper-Catalyzed Alkyne-Azide Cycloaddition

*Jin-Ping Gao, Fu-Qiang Zhang and Xian-Ming Zhang**

**Supporting Information**

# A 66-Nuclear All-Alkynyl Protected Peanut-Shaped Silver(I)/Copper(I) Heterometallic Nanocluster: Intermediate in Copper-Catalyzed Alkyne-Azide Cycloaddition

Jin-Ping Gao[†], Fu-Qiang Zhang[†], and Xian-Ming Zhang[*,†,§]

[†] *Key Laboratory of Magnetic Molecules & Magnetic Information Materials (Ministry of Education), School of Chemistry & Material Science, Shanxi Normal University, Taiyuan 030032, P. R. China.*

[§] *College of Chemistry, Taiyuan University of Technology, Taiyuan 030024, P. R. China.*

Corresponding Author

*E-mail: zhangxm@sxnu.edu.cn.

# Content


## 1. Experiment section

## 2. Supporting Figures

**Figure S1.** The molecule packing diagrams of **$Ag_{54}Cu_{12}$** in unit cell from b and c directions.

**Figure S2.** The coordination motifs of PhC≡$C^-$ with Ag and Cu atoms in **$Ag_{54}Cu_{12}$** cluster.

**Figure S3.** (a) Arrangement of $Mo_4O_{16}^{8-}$ units in **$Ag_{54}Cu_{12}$** cluster. (b) The polyhedral configuration of $Mo_4O_{16}$. (c) Side view of two $Mo_4O_{16}$ units fully overlapping and mutually twisted 24°.

**Figure S4.** The PXRD pattern of **$Ag_{54}Cu_{12}$**.

**Figure S5.** The IR spectra of **$Ag_{54}Cu_{12}$**.

**Figure S6.** SEM and EDS-Mapping images of **$Ag_{54}Cu_{12}$**.

**Figure S7.** (a) XPS survey spectrum of **$Ag_{54}Cu_{12}$**. Corresponding high-resolution XPS spectra in the (b) Ag 3d, (c) Mo 3d, and (d) Cu 2p regions.

**Figure S8.** The Cu MML Auger spectrum of **$Ag_{54}Cu_{12}$**.

**Figure S9.** Frontier molecular orbitals of **$Ag_{54}Cu_{12}$**: HOMO-1, HOMO-2, HOMO-3, and HOMO-4.

**Figure S10.** Mott-Schottky plot of $(PhC{\equiv}CAg)_n$ in a 0.2 M $Na_2SO_4$ aqueous solution.

**Figure S11.** The TEM images of **$Ag_{54}Cu_{12}$** loading with active carbon.

**Figure S12.** (a) The crystallization process of the product in the system, (b) Cycle stability, and (c) IR spectra of the catalyst before and after the reaction.

## 3. Supporting Tables

**Table S1.** Crystal data and structure refinement for **$Ag_{54}Cu_{12}$**.

**Table S2.** Bond Valence Sum (BVS) calculations for **$Ag_{54}Cu_{12}$** of $[Mo_4O_{16}]^{8-}$.

**Table S3.** The connection of between in two POMs of reported Ag nanoclusters.

**Table S4.** Selected bond lengths [Å] and angles [°] for **$Ag_{54}Cu_{12}$**.

**Table S5.** The crystal data of production under **$Ag_{54}Cu_{12}$**.

**Table S6.** The crystal data of production under **$Ag_{54}Cu_{12}$** as catalyst.

## 4. Supporting references

# 1. Experiment section

## 1.1 Crystallographic studies.

Single crystal of **$Ag_{54}Cu_{12}$** with appropriate dimensions was chosen under an optical microscope and quickly coated with high vacuum grease (Dow Corning Corporation) to prevent decomposition. Crystal was mounted on CryoLoop™ loop and the cell parameter and intensity data were recorded on a Rigaku Oxford Diffraction XtaLAB Synergy-S diffractometer equipped with a HyPix-6000HE Hybrid Photon Counting (HPC) detector operating in shutterless mode and an Oxford Cryosystems Cryostream 800 Plus at 150 K using Cu Kα (λ = 1.54184 Å) for **$Ag_{54}Cu_{12}$** from PhotonJet micro-focus X-ray Source. Data were processed using the CrystAlisPro software suite. Absorption corrections were applied by using the program CrysAlisPro (multi-scan).[S1] Crystal structure was examined using the Addsym subroutine of PLATON to ensure that no additional symmetry could be applied to the models. The structure was solved with direct methods and refined using Full-matrix least-squares based on $F_2$ with program SHELXL-97 within OLEX2.[S2]

This text discusses the synthesis of **$Ag_{54}Cu_{12}$** using an anion-templated and Cu reduction strategy with alkyne and phosphine. The role of phosphine was explored by comparing the selection or removal of phosphine ligands through changing their types as a control experiment. However, both experiments resulted in the crystallization of **$Ag_{54}Cu_{12}$** without producing any new species. Our extensive experimental results show that only the organic copper salt copper trifluoroacetate and $Cu(BF_4)_2$ produced satisfactory results, while other salts such as $CuCl_2$ and $Cu(SO_3CF_3)_2$ did not. We also discuss the conditions for catalytic reduction of several types of divalent copper salts to CuI-alkynes in the CuAAC reaction. Amines are necessary for the synthesis of copper halides at high temperatures without alkali or under ultrasonic conditions. The choice of solvent can also have an impact. To account for the solvent preference of silver, the precursor used is acetylene silver and a mixed solvent of methanol and *N*,*N*-dimethylformamide (DMF) is selected. Taking advantage of the weak reducibility in the synthesis, the addition of DMF can improve reduction efficiency, as reported in analogous silver nanoclusters.

### 1.2 Physical measurements.

Electrospray ionization–time of flight–mass spectrometry (ESI-TOF-MS) spectrometer was tested by Agilent Infinity II 6224-6230 Series TOF. Data was acquired using the following setting: ESI capillary voltage was set to 4000 V (positive mode) and fragmentor was set to 200 V. The liquid nebulizer was set to 15 psig and the nitrogen drying gas was set to a flow rate of 3 L/min. The mass spectra data analyses were conducted using Agilent MassHunter Workstation Data acquisition software (Version B.05.00) based on the isotope distribution patterns. UV-Vis absorption spectra were recorded at room temperature using TU-1950 UV-Vis spectrophotometer. The optical band gap was evaluated as a function of the Kubelka-Munk equation: $\sigma/S = (1-R)^2/2R$. Fourier-transform infrared (FTIR) spectra were obtained using a FTIR spectrophotometer (Thermo Nicolet 360). The X-ray photoelectron spectrum (XPS) spectra were obtained using the Thermo Scientific K-Alpha+ XPS with a monochromatic Al Ka X-ray source (1486.6 eV) operating at 72W (12kV, 6mA). The binding energies were referred to the C 1s peak of adventitious carbon at 284.8 eV. EDS-Mapping was obtained using the JSM-7500F. The photocurrent test and Mott-Schottky plot were carried out on a CHI660E electrochemistry workstation. 5 mg samples of 1-naphthol (5 wt. %, 10 mL) were dispersed in 80 mL of ethanol. The mixture was then subjected to ultrasound for 30 minutes. The resulting solution was transferred onto cleaned ITO glass using pipette tips. The coated film was obtained after evaporation at room temperature. The prepared ITO glass film was used as the working electrode, a Pt sheet as the counter electrode, and an Ag/AgCl electrode as the reference electrode. The medium used was 0.2 M $Na_2SO_4$ aqueous. In the testing process, we used $Ag_{54}Cu_{12}$-modified ITO glass as the working electrode, platinum wire as the assisting electrode, and Ag/AgCl (3 M KCl) as the reference electrode. The measurement condition involved maintaining a bias voltage of 0.6 V and a sensitivity of $10^{-5}$ for light irradiation at intervals of 20 seconds.

### 1.3 Synthesis.

**Synthesis of $Ag_{54}Cu_{12}$:** $(PhC{\equiv}CAg)_n$ precursor was prepared by reacting equivalent amounts of $Ag_2O$ and (PhC≡CH) according to previously reported procedures.[S3] The mixture of $(PhC{\equiv}CAg)_n$ (0.0021 g, 0.1 mmol) and $Cu(BF_4)_2$ (0.0130 g, 0.05 mmol) or $Cu(COOCF_3)_2$ (0.0080 g, 0.033 mmol) were dissolved in methanol and *N,N*-Dimethylformamide (7:1, 4 mL) under stirring. After 30 minutes, copper powder (0.0030 g, 0.025 mmol) and $Na_2MoO_4$ (0.001 g, 0.006 mmol) were added to the above solution and stirred continuously for 24 hours. Finally, the reaction mixture was sealed

and heated at 65°C for 20 hours. The solution slowly crystallized in glass bottle at 65℃ over the course of 7 days. A yield of 45% (10.8 mg, based on Ag) was obtained for the collection of a red block crystal of **$Ag_{54}Cu_{12}$**.

**Preparation of $C/TiO_2$-supported nanocluster catalysts:** A suspension was formed by dissolving 100 mg of activated charcoal in 10 mL of ethanol solution. Next, 1 mg of cluster was completely dissolved in 2 mL of methylene chloride solution to form a clarified brownish yellow solution. The above suspension was then slowly added drop by drop to the solution and stirred at room temperature for 12 hours until the upper solution was clarified, indicating that the clusters were fully loaded onto the $C/TiO_2$ support. The nanocatalysts loaded with clusters were collected through centrifugation at 10,000 rpm for 3 minutes and then vacuum-dried overnight.

### 1.4 Procedure for cycloaddition reactions

Benzyl azide (25 $\mu$L, 0.2 mmol), phenylacetylene (24 $\mu$L, 0.22 mmol), and 1 mL $CH_3CN$ were added in a 25 mL round bottom flask. 5 mg **$Ag_{54}Cu_{12}$** and $C/TiO_2$-supported nanocluster catalysts were added to reaction as catalysts. The reaction was carried out at 40°C for 12 h. The organic layer containing the product was separated, and the solids were washed with dichloromethane (2 × 3 mL). The solvent in the combined organic solutions was removed by rotary evaporation, followed by purification using column chromatography (silica gel, dichloromethane or 1/1 dichloromethane/ethylacetate).

For the recycling experiments, the solid catalyst recovered via centrifugation was dried at room temperature under vacuum overnight, before being used in the next run.

## 2. Supporting Figures

**Figure S1.** The molecule packing diagrams of $\mathbf{Ag_{54}Cu_{12}}$ in unit cell from (a) b and (b) c directions.

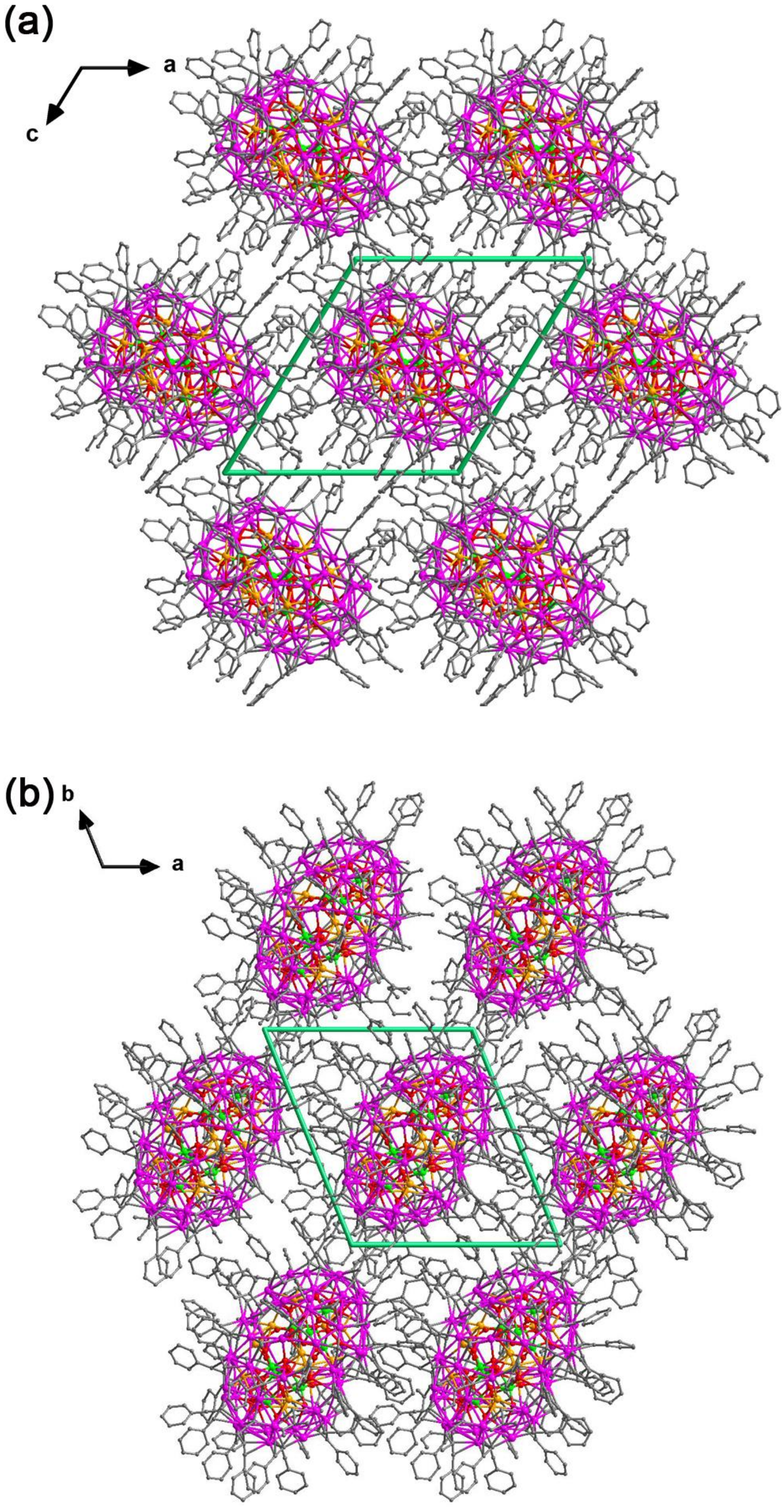

**Figure S2.** The coordination motifs of PhC≡C$^-$ with Ag and Cu atoms in **Ag$_{54}$Cu$_{12}$** cluster.

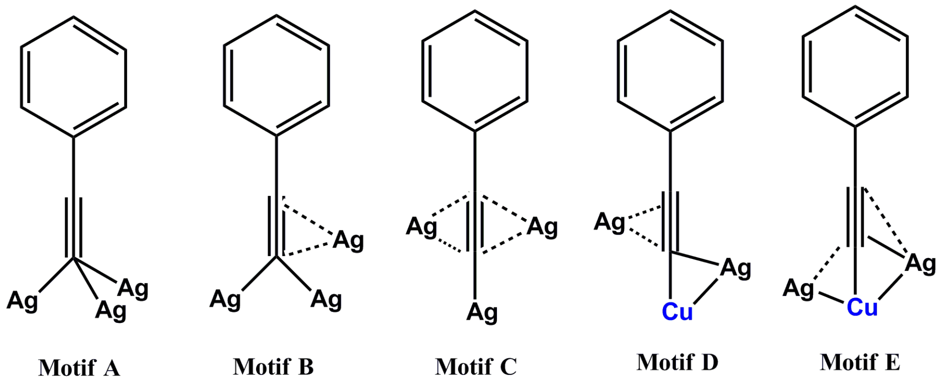


**Figure S3.** (a) Arrangement of Mo$_4$O$_{16}$$^{8-}$ units in **Ag$_{54}$Cu$_{12}$** cluster. (b) The polyhedral configuration of Mo$_4$O$_{16}$. (c) Side view of two Mo$_4$O$_{16}$ units fully overlapping and mutually twisted 24°.

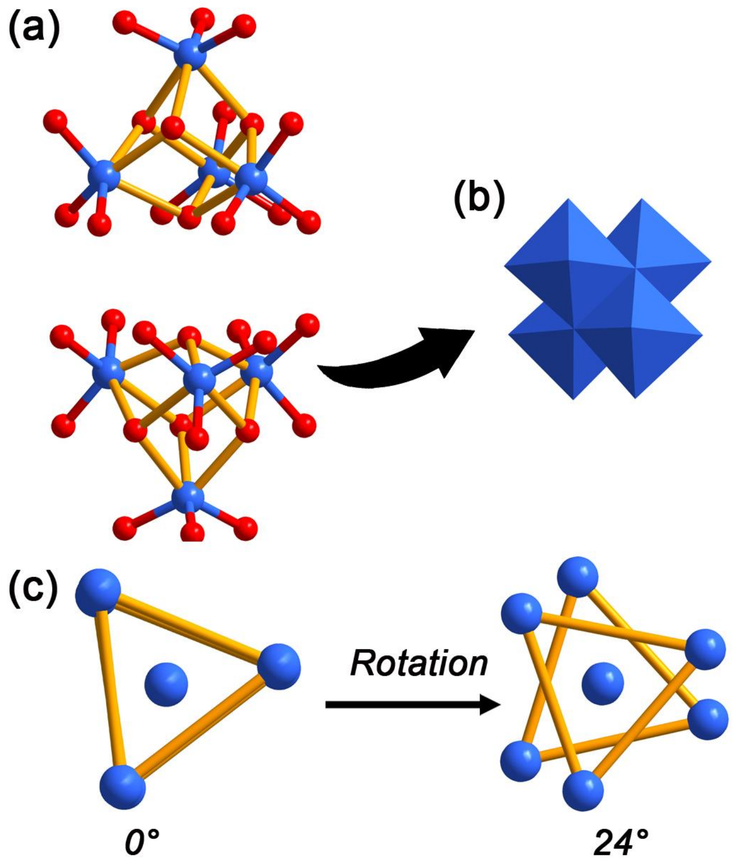

## 2.2 The composition analysis of $Ag_{54}Cu_{12}$.

**Figure S4.** The PXRD pattern of $\mathbf{Ag_{54}Cu_{12}}$.

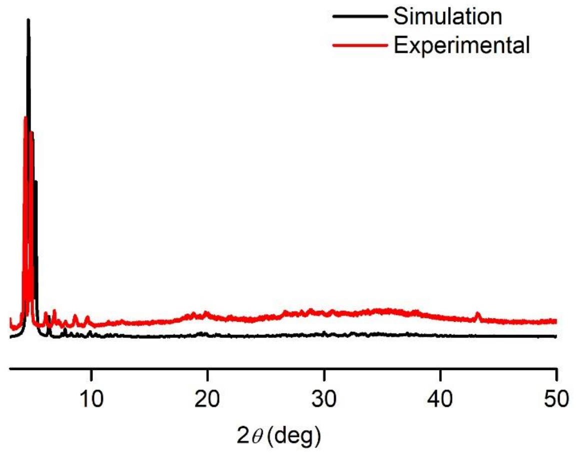


**Figure S5.** The IR spectra of $\mathbf{Ag_{54}Cu_{12}}$.

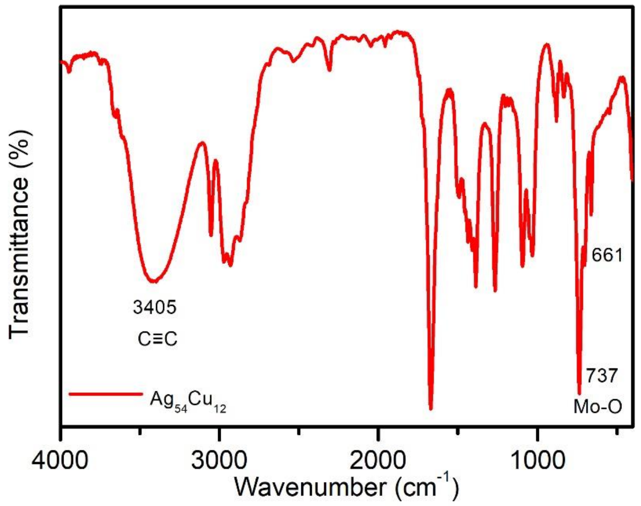


**Figure S6.** SEM and EDS-Mapping images of $\mathbf{Ag_{54}Cu_{12}}$.

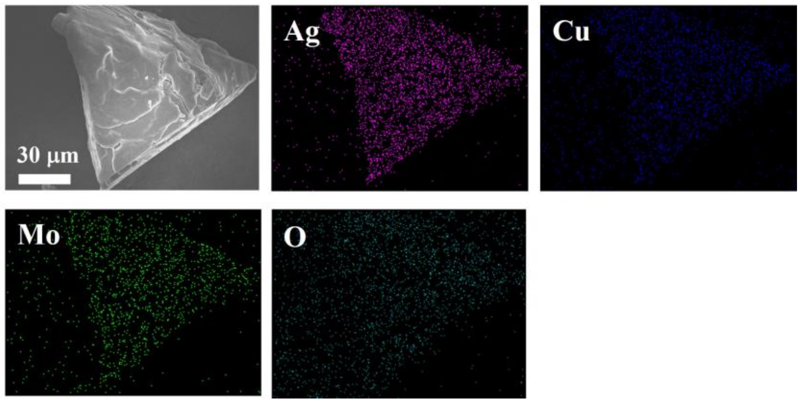

**Figure S7.** (a) XPS survey spectrum of $\mathbf{Ag_{54}Cu_{12}}$. Corresponding high-resolution XPS spectra in the (b) Ag 3d, (c) Mo 3d, and (d) Cu 2p regions.

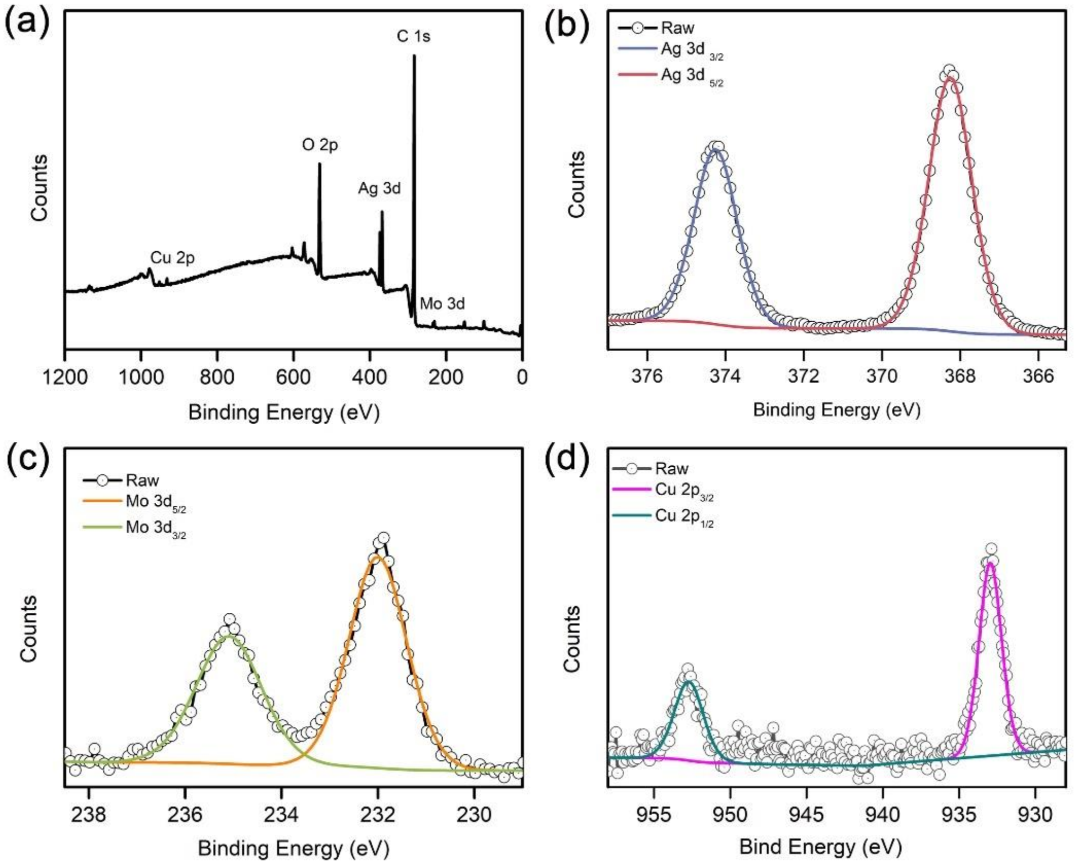


**Figure S8.** The Cu MML Auger spectrum of $\mathbf{Ag_{54}Cu_{12}}$.

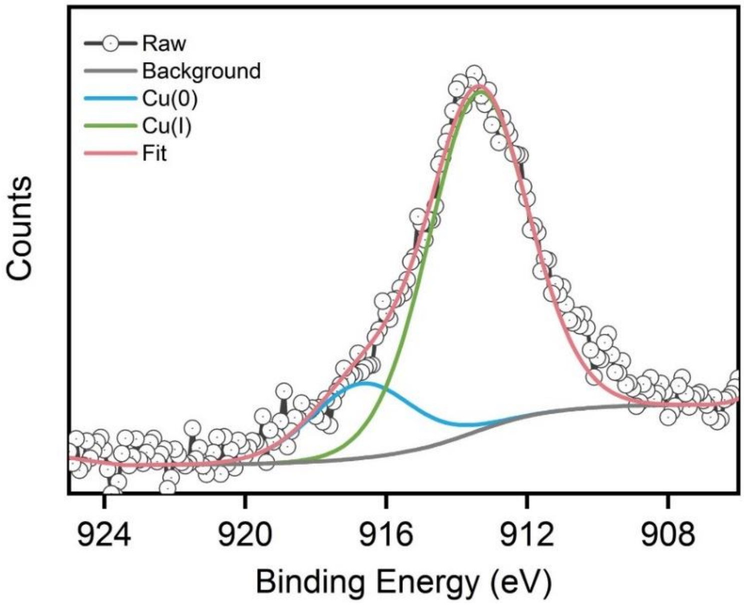

**Figure S9.** Frontier molecular orbitals of **$Ag_{54}Cu_{12}$**: HOMO-1, HOMO-2, HOMO-3, and HOMO-4.

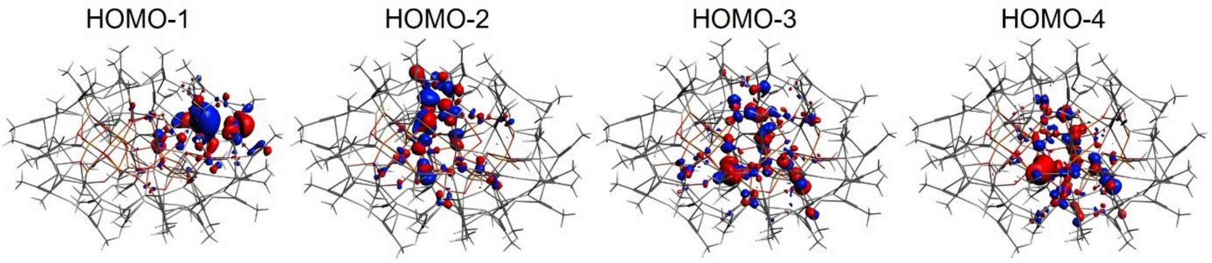


## 2.3 The physical properties.

**Figure S10.** Mott–Schottky plot of $(PhC{\equiv}CAg)_n$ in a 0.2 M $Na_2SO_4$ aqueous solution.

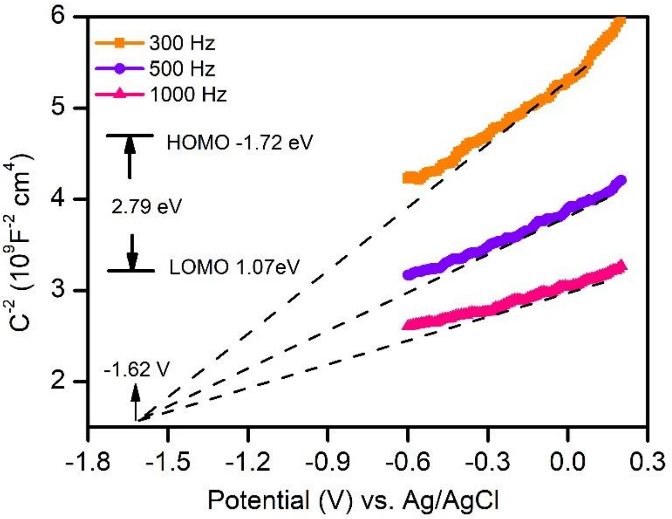


## 2.4 The characterize of $Ag_{54}Cu_{12}$/C

**Figure S11.** The TEM images of **$Ag_{54}Cu_{12}$** loading with active carbon.

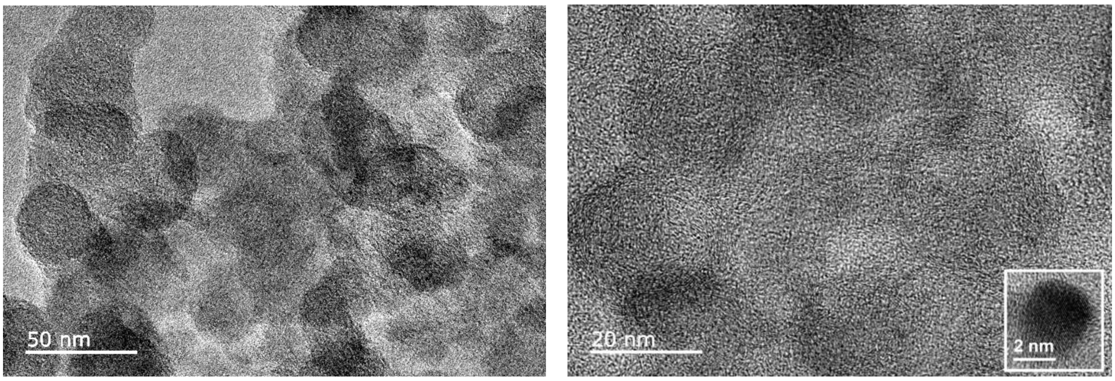

## 3. Supporting tables

**Table S1.** Crystal data and structure refinement for **$Ag_{54}Cu_{12}$**.

| Compound | **$Ag_{54}Cu_{12}$** |
|---|---|
| Empirical formula | $C_{400}H_{250}Ag_{54}Cu_{12}Mo_8O_{32}$ |
| Formula weight | 12922.96 |
| Temperature | 150.00(10) |
| Crystal system | triclinic |
| Space group | $P1$ |
| $a$ (Å) | 21.62055(10) |
| $b$ (Å) | 22.33187(10) |
| $c$ (Å) | 23.42304(9) |
| α (°) | 104.2338(4) |
| $\beta$ (°) | 116.1709(4) |
| $\gamma$ (°) | 102.6127(4) |
| $V$ (Å$^3$) | 9116.79(7) |
| $Z$ | 1 |
| $\rho_{calc}$ (g cm$^{-3}$) | 2.354 |
| $\mu$ ($mm^{-1}$) | 26.008 |
| $F$ (000) | 6128.0 |
| Size (mm) | 0.15×0.15×0.15 |
| Reflections | 294926 |
| Data/parameters | 62554/2996 |
| $R_1$[a], w$R_2$[b] [$I$>$2\sigma$(I)] | 0.0843/0.2205 |
| $R_1$[a], w$R_2$[b] (all data) | 0.0851/0.2214 |
| $\Delta\rho_{max}/\Delta\rho_{min}$(eÅ$^{-3}$) | 2.77/-3.21 |
| Flack parameter | 0.094(9) |

[a]$R_1 = \Sigma||F_o| - |F_c||/\Sigma|F_o|$. [b]$wR_2 = \{\Sigma[w(F_o^2 - F_c^2)^2]/\Sigma[w(F_0^2)^2]\}^{1/2}$

**Table S2.** Bond Valence Sum (BVS) calculations for $\mathbf{Ag_{54}Cu_{12}}$ of $[Mo_4O_{16}]^{8-}$.

| Bond | | D | BVS | Bond | | D | BVS | Bond | | D | BVS |
|---|---|---|---|---|---|---|---|---|---|---|---|
| Mo1 | O1 | 1.757 | 1.49989 | Mo2 | O4 | 1.770 | 1.44601 | Mo3 | O5 | 2.128 | 0.55029 |
| Mo1 | O2 | 1.754 | 1.50825 | Mo2 | O5 | 2.217 | 0.43188 | Mo3 | O6 | 1.761 | 1.48179 |
| Mo1 | O3 | 1.782 | 1.40089 | Mo2 | O9 | 2.109 | 0.57774 | Mo3 | O7 | 2.184 | 0.47223 |
| Mo1 | O5 | 2.196 | 0.45786 | Mo2 | O11 | 1.793 | 1.36053 | Mo3 | O10 | 1.769 | 1.44869 |
| Mo1 | O7 | 2.197 | 0.45632 | Mo2 | O12 | 1.747 | 1.53952 | Mo3 | O13 | 2.151 | 0.51668 |
| Mo1 | O9 | 2.185 | 0.47156 | Mo2 | O13 | 2.145 | 0.52483 | Mo3 | O14 | 1.785 | 1.38858 |
| | | | **5.79480** | | | | **5.88053** | | | | **5.85828** |
| Mo4 | O9 | 2.187 | 0.46852 | Mo5 | O17 | 1.772 | 1.43830 | Mo6 | O23 | 1.755 | 1.50692 |
| Mo4 | O7 | 2.113 | 0.57245 | Mo5 | O21 | 2.248 | 0.39711 | Mo6 | O24 | 1.771 | 1.44198 |
| Mo4 | O8 | 1.753 | 1.51331 | Mo5 | O18 | 1.740 | 1.56640 | Mo6 | O21 | 2.258 | 0.38672 |
| Mo4 | O16 | 1.791 | 1.36585 | Mo5 | O27 | 2.197 | 0.45622 | Mo6 | O29 | 1.762 | 1.47668 |
| Mo4 | O13 | 2.165 | 0.49791 | Mo5 | O22 | 1.747 | 1.53873 | Mo6 | O27 | 2.119 | 0.56354 |
| Mo4 | O15 | 1.763 | 1.47185 | Mo5 | O26 | 2.101 | 0.59044 | Mo6 | O31 | 2.170 | 0.49104 |
| | | | **5.88992** | | | | **5.98722** | | | | **5.86690** |
| Mo7 | O21 | 2.246 | 0.39980 | Mo8 | O27 | 2.180 | 0.47788 | | | | |
| Mo7 | O20 | 1.768 | 1.45478 | Mo8 | O31 | 2.192 | 0.46215 | | | | |
| Mo7 | O19 | 1.759 | 1.48956 | Mo8 | O26 | 2.194 | 0.46001 | | | | |
| Mo7 | O31 | 2.122 | 0.55805 | Mo8 | O30 | 1.727 | 1.62565 | | | | |
| Mo7 | O26 | 2.191 | 0.46383 | Mo8 | O28 | 1.754 | 1.51154 | | | | |
| Mo7 | O25 | 1.771 | 1.44110 | Mo8 | O32 | 1.760 | 1.48763 | | | | |
| | | | **5.80713** | | | | **6.02488** | | | | |

**Table S3.** The connection of between in two POMs of reported Ag nanoclusters.

| Name | Connection | Structure of cluster | Ref. |
|---|---|---|---|
| **$Ag_{60}$** | $Mo_6O_{22}$ connecting with Ag-Ag interaction | 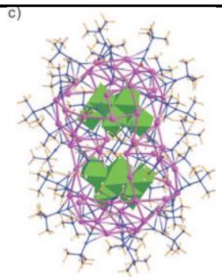  | *Angew. Chem. Int. Ed*. **2010**, 49, 1765-1767 |
| **$Ag_{90}$** | $W_5O_{19}$ connecting with two $SO_4^{2-}$ | 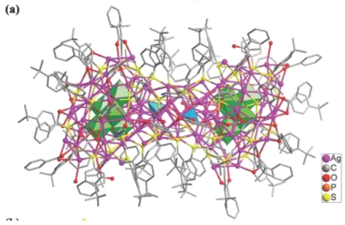  | *Chem. Commun*., **2018**, 54, 4461-4464 |
| **$Ag_{42}$** | $Eu(W_5O_{18})_2$ TM-substituted | 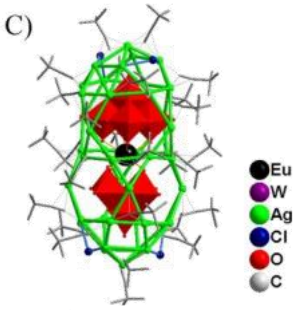  | *Dalton Trans*., **2015**, 44, 3997-4002 |
| **$Ag_{80}$** | $Mo_7O_{26}$ connecting with $Ag_{10}$ | 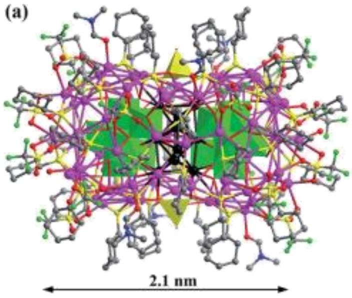  | *Chem. Sci*., **2019**, 10, 564 |
| **$Ag_{54}$** | $CO_3^{2-}$ connecting with $Ag_{11}$ | 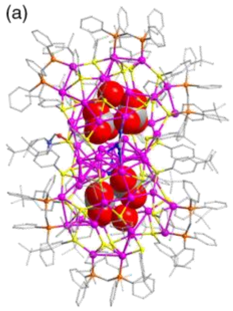  | *CCS Chem*., **2019**, 1, 663-672 |

| | | | |
|---|---|---|---|
| **$Ag_{84}$** | $W_7O_{26}$ connecting with $Ag_{10}$ | 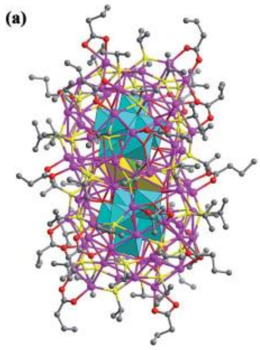  | *Chem. Sci.*, **2019**, 10, 4862-4867 |
| **$Ag_{72}$** | Two $EuW_{10}O_{36}$ connecting with $Ag_{11}$ | 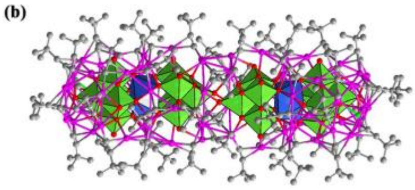  | *Chem. Eur. J.* **2018**, 24, 1998-2003 |
| **$Ag_{72}$** | $PW_9O_{34}$ The connection of Ag-O bond | 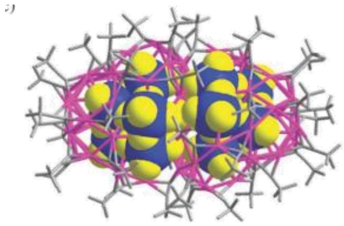 | *Chem. Commun.*, **2014**, 50, 2353-2355 |
| **$Ag_{54}Cu_{12}$** | $Mo_4O_{16}$ No linker | 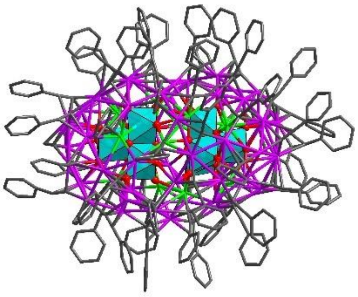 | *This work* |

**Table S4.** Selected bond lengths [Å] for **$Ag_{54}Cu_{12}$**.

| | **$Ag_{54}Cu_{12}$** | | |
|---|---|---|---|
| Ag1-Ag3 | 3.332(2) | Ag35-Ag43 | 3.054(4) |
| Ag1-Ag4 | 3.121(2) | Ag35-Cu10 | 2.876(3) |
| Ag1-Ag5 | 2.962(2) | Ag35-C42 | 2.359(17) |
| Ag1-Ag6 | 3.363(2) | Ag35-C110 | 2.25(2) |
| Ag1-Ag12 | 3.337(2) | Ag35-C362 | 2.36(4) |
| Ag1-O3 | 2.555(10) | Ag36-Ag45 | 3.158(2) |
| Ag1-C54 | 2.313(19) | Ag36-Cu7 | 2.888(3) |
| Ag1-C218 | 2.352(18) | Ag36-Cu10 | 2.860(3) |
| Ag1-C298 | 2.32(2) | Ag36-C12 | 2.090(17) |
| Ag2-Ag3 | 3.0590(18) | Ag36-C38 | 2.263(17) |
| Ag2-Ag6 | 3.2342(19) | Ag36-C250 | 2.53(2) |
| Ag2-Ag9 | 3.0552(19) | Ag37-Ag45 | 3.343(2) |
| Ag2-C66 | 2.248(19) | Ag37-O29 | 2.395(9) |
| Ag2-C172 | 2.604(18) | Ag37-C6 | 2.360(15) |
| Ag2-C298 | 2.14(2) | Ag37-C12 | 2.424(17) |
| Ag3-Ag4 | 3.0914(19) | Ag37-C24 | 2.374(18) |
| Ag3-Ag9 | 3.173(2) | Ag37-C34 | 2.605(16) |
| Ag3-Ag10 | 3.296(3) | Ag37-C36 | 2.613(17) |
| Ag3-O1 | 2.560(11) | Ag37-C58 | 2.686(17) |
| Ag3-C198 | 2.48(2) | Ag38-Ag39 | 3.027(3) |
| Ag3-C258 | 2.32(2) | Ag38-C24 | 2.089(17) |
| Ag3-C298 | 2.25(2) | Ag38-C351 | 2.07(3) |
| Ag4-Ag5 | 3.061(2) | Ag39-Ag40 | 2.950(3) |
| Ag4-Ag11 | 2.912(2) | Ag39-Ag54 | 3.256(3) |
| Ag4-Cu2 | 2.843(3) | Ag39-Cu11 | 2.748(3) |
| Ag4-C40 | 2.536(17) | Ag39-C28 | 2.455(17) |
| Ag4-C54 | 2.305(19) | Ag39-C297 | 2.22(3) |
| Ag4-C258 | 2.18(2) | Ag39-C351 | 2.29(3) |
| Ag5-Ag12 | 3.106(2) | Ag39-Ag47 | 2.970(4) |
| Ag5-Ag19 | 3.1206(18) | Ag40-Ag41 | 3.142(3) |
| Ag5-C26 | 2.076(18) | Ag40-Ag54 | 3.204(3) |
| Ag5-C54 | 2.099(19) | Ag40-Cu11 | 3.002(3) |
| Ag6-Ag7 | 3.091(2) | Ag40-O25 | 2.517(11) |
| Ag6-Ag12 | 2.9243(17) | Ag40-C87 | 2.28(2) |
| Ag6-Cu3 | 2.857(3) | Ag40-C297 | 2.24(3) |
| Ag6-C30 | 2.574(15) | Ag40-Ag31 | 3.065(3) |
| Ag6-C172 | 2.284(19) | Ag40-Ag1B | 2.999(9) |
| Ag6-C218 | 2.131(18) | Ag41-Ag53 | 3.042(4) |
| Ag7-Ag8 | 3.1503(19) | Ag41-Ag54 | 3.313(3) |
| Ag7-Ag13 | 3.060(2) | Ag41-C228 | 2.02(3) |
| Ag7-C102 | 2.01(2) | Ag41-C344 | 2.05(3) |
| Ag7-C172 | 2.087(19) | Ag42-Ag43 | 3.199(4) |
| Ag8-Ag9 | 2.9134(18) | Ag42-Ag46 | 3.378(4) |
| Ag8-Ag14 | 3.0211(19) | Ag42-Ag53 | 2.981(4) |
| Ag8-Ag15 | 3.040(2) | Ag42-Cu12 | 2.904(4) |
| Ag8-Cu1 | 3.014(3) | Ag42-O28 | 2.593(11) |
| Ag8-O2 | 2.579(10) | Ag42-C177 | 2.27(3) |
| Ag8-O6 | 2.564(10) | Ag42-C349 | 2.51(4) |
| Ag8-C66 | 2.223(19) | Ag43-Ag44 | 2.892(3) |
| Ag8-C222 | 2.25(2) | Ag43-C362 | 2.04(4) |
| Ag9-Ag10 | 2.989(2) | Ag44-Ag45 | 2.906(3) |
| Ag9-Cu1 | 2.814(3) | Ag44-Ag46 | 3.139(4) |
| Ag9-C60 | 2.497(18) | Ag44-Cu10 | 2.909(3) |
| Ag9-C66 | 2.184(19) | Ag44-C42 | 2.649(17) |
| Ag9-C198 | 2.30(2) | Ag44-C252 | 2.21(3) |
| Ag10-Ag11 | 3.066(2) | Ag44-Ag1A | 2.888(6) |

| | | | |
|---|---|---|---|
| Ag10-Ag16 | 3.118(3) | Ag45-Cu10 | 2.892(3) |
| Ag10-C198 | 2.03(2) | Ag45-C12 | 2.297(17) |
| Ag10-C246 | 2.05(3) | Ag45-C252 | 2.19(3) |
| Ag11-Ag17 | 2.854(2) | Ag45-Ag1A | 3.239(5) |
| Ag11-Ag18 | 3.0622(19) | Ag46-Ag53 | 3.144(4) |
| Ag11-Cu2 | 3.152(3) | Ag46-O28 | 2.544(12) |
| Ag11-O4 | 2.550(11) | Ag46-C349 | 2.25(4) |
| Ag11-C108 | 2.264(18) | Ag46-C353 | 2.34(3) |
| Ag11-C258 | 2.24(2) | Ag46-C383 | 2.24(7) |
| Ag12-Ag20 | 2.9677(17) | Ag46-Ag1A | 2.984(5) |
| Ag12-Ag21 | 3.0603(19) | Ag48-Ag51 | 3.3634(18) |
| Ag12-Cu3 | 2.959(3) | Ag48-Ag52 | 2.9970(17) |
| Ag12-O3 | 2.550(11) | Ag48-O15 | 2.479(9) |
| Ag12-C62 | 2.255(19) | Ag48-O20 | 2.529(9) |
| Ag12-C218 | 2.263(18) | Ag48-C22 | 2.240(17) |
| Ag13-Ag14 | 3.126(2) | Ag48-C138 | 2.301(18) |
| Ag13-Ag22 | 2.871(2) | Ag49-Ag51 | 3.0807(17) |
| Ag13-Cu3 | 2.805(3) | Ag49-Cu6 | 2.739(2) |
| Ag13-C30 | 2.339(16) | Ag49-Cu9 | 2.783(2) |
| Ag13-C68 | 2.255(18) | Ag49-C8 | 2.704(16) |
| Ag13-C102 | 2.41(2) | Ag49-C48 | 2.614(16) |
| Ag14-Ag22 | 3.016(2) | Ag49-C52 | 2.330(17) |
| Ag14-O6 | 2.525(9) | Ag49-C68 | 2.362(19) |
| Ag14-C70 | 2.315(19) | Ag49-C104 | 2.595(18) |
| Ag14-C102 | 2.39(2) | Ag49-C248 | 2.63(2) |
| Ag14-C222 | 2.35(2) | Ag50-Ag52 | 3.0598(14) |
| Ag14-C318 | 2.71(3) | Ag50-Cu5 | 2.735(2) |
| Ag14-C388 | 2.65(3) | Ag50-Cu8 | 2.789(2) |
| Ag15-Cu1 | 2.785(3) | Ag50-C1 | 2.325(14) |
| Ag15-Cu4 | 2.886(3) | Ag50-C4 | 2.643(14) |
| Ag15-C170 | 2.463(19) | Ag50-C10 | 2.683(14) |
| Ag15-C202 | 2.290(17) | Ag50-C14 | 2.319(16) |
| Ag15-C222 | 2.12(2) | Ag50-C16 | 2.620(15) |
| Ag16-Ag17 | 3.288(3) | Ag50-C32 | 2.638(16) |
| Ag16-Ag26 | 2.898(2) | Ag51-Cu6 | 3.056(3) |
| Ag16-Cu1 | 2.812(3) | Ag51-C8 | 2.596(16) |
| Ag16-C56 | 2.265(19) | Ag51-C52 | 2.110(16) |
| Ag16-C60 | 2.386(18) | Ag51-C138 | 2.075(19) |
| Ag16-C246 | 2.32(3) | Ag51-C363 | 2.59(9) |
| Ag17-Ag18 | 3.233(3) | Ag51-Ag31 | 3.008(2) |
| Ag17-Ag26 | 3.094(2) | Ag51-Ag1B | 3.168(10) |
| Ag17-O4 | 2.488(9) | Ag52-Cu8 | 2.965(2) |
| Ag17-C64 | 2.280(19) | Ag52-C4 | 2.570(14) |
| Ag17-C88 | 2.615(19) | Ag52-C14 | 2.139(16) |
| Ag17-C108 | 2.250(18) | Ag52-C22 | 2.147(17) |
| Ag17-C246 | 2.66(3) | Ag53-Ag54 | 3.241(4) |
| Ag17-C272 | 2.67(3) | Ag53-Cu12 | 2.813(3) |
| Ag18-Cu2 | 2.734(3) | Ag53-C69 | 2.68(6) |
| Ag18-Cu5 | 2.839(3) | Ag53-C84 | 2.528(18) |
| Ag18-C10 | 2.281(13) | Ag53-C344 | 2.31(3) |
| Ag18-C18 | 2.574(14) | Ag53-C349 | 2.07(4) |
| Ag18-C108 | 2.130(18) | Ag54-O32 | 2.517(11) |
| Ag19-Ag20 | 3.2222(18) | Ag54-C297 | 2.14(3) |
| Ag19-Ag52 | 2.9281(17) | Ag54-C344 | 2.40(3) |
| Ag19-Cu2 | 2.778(3) | Ag54-C353 | 2.20(3) |
| Ag19-C14 | 2.299(16) | Ag54-Ag47 | 3.109(4) |
| Ag19-C26 | 2.353(18) | Mo1-O1 | 1.758(10) |
| Ag19-C40 | 2.393(17) | Mo1-O2 | 1.755(9) |
| Ag20-Ag21 | 3.238(2) | Mo1-O3 | 1.781(11) |
| Ag20-Ag52 | 3.1411(16) | Mo1-O5 | 2.197(10) |

| | | | |
|---|---|---|---|
| Ag20-O8 | 2.464(10) | Mo1-O7 | 2.198(9) |
| Ag20-C22 | 2.325(17) | Mo1-O9 | 2.185(9) |
| Ag20-C26 | 2.480(18) | Mo2-Cu1 | 3.112(2) |
| Ag20-C62 | 2.261(18) | Mo2-O4 | 1.770(10) |
| Ag20-C226 | 2.601(19) | Mo2-O5 | 2.206(8) |
| Ag21-Cu3 | 2.774(3) | Mo2-O9 | 2.110(9) |
| Ag21-Cu6 | 2.854(3) | Mo2-O11 | 1.794(10) |
| Ag21-C8 | 2.250(16) | Mo2-O12 | 1.746(9) |
| Ag21-C62 | 2.152(19) | Mo2-O13 | 2.146(8) |
| Ag21-C200 | 2.49(2) | Mo3-O5 | 2.127(9) |
| Ag22-Ag23 | 3.285(2) | Mo3-O6 | 1.761(9) |
| Ag22-Ag49 | 3.1095(18) | Mo3-O7 | 2.184(8) |
| Ag22-Cu9 | 2.983(3) | Mo3-O10 | 1.770(9) |
| Ag22-C48 | 2.599(16) | Mo3-O13 | 2.151(8) |
| Ag22-C68 | 2.100(19) | Mo3-O14 | 1.785(9) |
| Ag22-C70 | 2.06(2) | Mo4-O7 | 2.112(8) |
| Ag23-Ag24 | 3.274(2) | Mo4-O8 | 1.754(9) |
| Ag23-O10 | 2.474(9) | Mo4-O9 | 2.187(8) |
| Ag23-O18 | 2.471(10) | Mo4-O13 | 2.165(8) |
| Ag23-C70 | 2.295(19) | Mo4-O15 | 1.764(9) |
| Ag23-C360 | 2.29(3) | Mo4-O16 | 1.791(9) |
| Ag24-Ag25 | 3.090(2) | Mo5-O17 | 1.772(10) |
| Ag24-Ag34 | 3.180(3) | Mo5-O18 | 1.742(9) |
| Ag24-Ag35 | 2.842(2) | Mo5-O21 | 2.249(8) |
| Ag24-Cu4 | 2.916(3) | Mo5-O22 | 1.746(11) |
| Ag24-C110 | 2.11(2) | Mo5-O26 | 2.103(10) |
| Ag24-C202 | 2.512(17) | Mo5-O27 | 2.196(9) |
| Ag24-C360 | 2.02(3) | Mo6-Cu11 | 3.107(2) |
| Ag25-Ag26 | 2.997(2) | Mo6-O21 | 2.260(8) |
| Ag25-Cu4 | 2.772(2) | Mo6-O23 | 1.755(9) |
| Ag25-Cu7 | 2.800(3) | Mo6-O24 | 1.770(9) |
| Ag25-C38 | 2.612(17) | Mo6-O27 | 2.120(9) |
| Ag25-C56 | 2.375(19) | Mo6-O29 | 1.763(9) |
| Ag25-C110 | 2.37(2) | Mo6-O31 | 2.169(9) |
| Ag25-C202 | 2.575(17) | Mo7-O19 | 1.760(9) |
| Ag25-C300 | 2.67(2) | Mo7-O20 | 1.767(9) |
| Ag26-Ag27 | 3.2248(19) | Mo7-O21 | 2.247(8) |
| Ag26-Cu7 | 3.003(3) | Mo7-O25 | 1.773(10) |
| Ag26-C38 | 2.643(17) | Mo7-O26 | 2.191(10) |
| Ag26-C56 | 2.12(2) | Mo7-O31 | 2.124(8) |
| Ag26-C64 | 2.09(2) | Mo8 O26 | 2.195(9) |
| Ag27-Ag28 | 3.1734(16) | Mo8-O27 | 2.180(10) |
| Ag27-O12 | 2.464(9) | Mo8-O28 | 1.755(11) |
| Ag27-O23 | 2.464(9) | Mo8-O30 | 1.728(11) |
| Ag27-C6 | 2.250(16) | Mo8-O31 | 2.194(10) |
| Ag27-C64 | 2.276(19) | Mo8-O32 | 1.761(11) |
| Ag28-Ag29 | 2.8722(16) | Cu1-O5 | 1.855(9) |
| Ag28-Ag37 | 3.1241(15) | Cu1-C60 | 1.849(19) |
| Ag28-Ag50 | 3.0625(14) | Cu2-O9 | 1.875(9) |
| Ag28-Cu5 | 3.008(2) | Cu2-C40 | 1.838(17) |
| Ag28-C1 | 2.163(15) | Cu3-O7 | 1.881(9) |
| Ag28-C6 | 2.120(16) | Cu3-C30 | 1.843(16) |
| Ag28-C10 | 2.589(13) | Cu4-Cu7 | 2.893(3) |
| Ag29-Ag37 | 3.1445(16) | Cu4-O10 | 2.070(9) |
| Ag29 Ag38 | 3.1151(17) | Cu4-O11 | 1.929(9) |
| Ag29-Cu11 | 2.942(2) | Cu4-O17 | 2.292(11) |
| Ag29-C1 | 2.250(15) | Cu4-C202 | 1.856(18) |
| Ag29-C24 | 2.422(17) | Cu5-Cu8 | 2.933(3) |
| Ag29-C28 | 2.383(17) | Cu5-O12 | 2.083(9) |
| Ag29-C46 | 2.694(16) | Cu5-O16 | 1.920(9) |

| | | | |
|---|---|---|---|
| Ag30-Ag40 | 3.151(2) | Cu5-C10 | 1.888(13) |
| Ag30-Cu8 | 2.946(2) | Cu6-Cu9 | 2.914(3) |
| Ag30-Cu11 | 2.824(3) | Cu6-O14 | 1.919(9) |
| Ag30-C4 | 2.272(14) | Cu6-O15 | 2.092(9) |
| Ag30-C87 | 2.13(2) | Cu6-C8 | 1.842(17) |
| Ag30-C156 | 2.511(16) | Cu7-O11 | 2.429(11) |
| Ag30-Ag1B | 2.673(11) | Cu7-O17 | 1.959(9) |
| Ag32-Ag41 | 3.161(2) | Cu7-O23 | 2.063(9) |
| Ag32-Ag51 | 2.8647(19) | Cu7-C38 | 1.865(17) |
| Ag32-Cu12 | 2.885(3) | Cu8-O16 | 2.220(9) |
| Ag32-C52 | 2.247(16) | Cu8-O20 | 2.070(9) |
| Ag32-C84 | 2.372(19) | Cu8-O24 | 1.970(9) |
| Ag32-C228 | 2.40(3) | Cu8-C4 | 1.854(15) |
| Ag32-C363 | 1.05(9) | Cu9-O14 | 2.260(9) |
| Ag32-Ag31 | 3.134(3) | Cu9-O18 | 2.098(10) |
| Ag33-Ag42 | 3.063(3) | Cu9-O19 | 1.990(10) |
| Ag33-Cu9 | 2.929(3) | Cu9-C48 | 1.857(16) |
| Ag33-Cu12 | 2.807(4) | Cu10-O27 | 1.872(9) |
| Ag33-C48 | 2.276(16) | Cu10-C42 | 1.815(18) |
| Ag33-C106 | 2.522(19) | Cu11-O31 | 1.874(9) |
| Ag33-C177 | 2.14(3) | Cu11-C28 | 1.837(18) |
| Ag34-Ag35 | 3.225(3) | Cu12-O26 | 1.868(9) |
| Ag34-Ag42 | 3.015(4) | Cu12-C84 | 1.838(19) |
| Ag34-O22 | 2.441(10) | O25-Ag31 | 2.501(10) |
| Ag34-C177 | 2.31(3) | O25-Ag1B | 2.484(14) |
| Ag34-C207 | 2.67(4) | O30-Ag47 | 2.432(11) |
| Ag34-C360 | 2.38(3) | | |
| Ag34-C362 | 2.29(4) | | |
| Ag34-C390 | 2.58(3) | | |

**Table S5.** The crystal data of production under **$Ag_{54}Cu_{12}$**.

| Compound | 1-benzyl-4-phenyl-1H-1,2,3-triazole | 1-benzyl-4-(4-nitrophenyl)-1H-1,2,3-triazole | 1-benzyl-4-(4-methoxyphenyl)-1H-1,2,3-triazole |
|---|---|---|---|
| Empirical formula | $C_{15}H_{13}N_3$ | $C_{15}H_{12}N_4O_2$ | $C_{16}H_{15}N_3O$ |
| Formula weight | 235.28 | 280.29 | 266.32 |
| Temperature | 149.99(10) | 293(2) | 293(2) |
| Crystal system | monoclinic | monoclinic | monoclinic |
| Space group | $P2_1/c$ | $P2_1/n$ | $P2_1$ |
| $a$ (Å) | 6.00380(10) | 5.79470(10) | 8.15320(10) |
| $b$ (Å) | 8.04660(10) | 18.4036(3) | 5.68450(10) |
| $c$ (Å) | 25.2031(3) | 12.6719(2) | 14.7608(2) |
| α (°) | 90 | 90 | 90 |
| $\beta$ (°) | 94.3790(10) | 98.043(2) | 93.7330(10) |
| $\gamma$ (°) | 90 | 90 | 90 |
| $V$ (Å$^3$) | 1214.01(3) | 1338.08(4) | 682.665(17) |
| $Z$ | 4 | 4 | 2 |
| $\rho_{calc}$ (g cm$^{-3}$) | 1.287 | 1.391 | 1.296 |
| $\mu$ ($mm^{-1}$) | 0.618 | 0.794 | 0.666 |
| $F$ (000) | 496.0 | 584.0 | 282.0 |
| Size (mm) | 0.12 × 0.1 × 0.08 | 0.1 × 0.08 × 0.05 | 0.08 × 0.08 × 0.05 |
| Reflections | 9935 | 6545 | 12132 |
| Data / parameters | 2469/164 | 2648/190 | 2716/182 |
| $R_1$[a], w$R_2$[b] [$I>2\sigma$(I)] | 0.0390/0.1003 | 0.0363/0.0979 | 0.0315/0.0943 |
| $R_1$[a], w$R_2$[b] (all data) | 0.0411/0.1015 | 0.0409/0.1018 | 0.0322/0.0953 |
| $\Delta\rho_{max}/\Delta\rho_{min}$(eÅ$^{-3}$) | 0.25/-0.16 | 0.10/-0.15 | 0.10/-0.25 |
| Flack parameter | — | — | 0.12(9) |

**Figure S12.** (a) The crystallization process of the product in the system, (b) Cycle stability, and (c) IR spectra of the catalyst before and after the reaction.

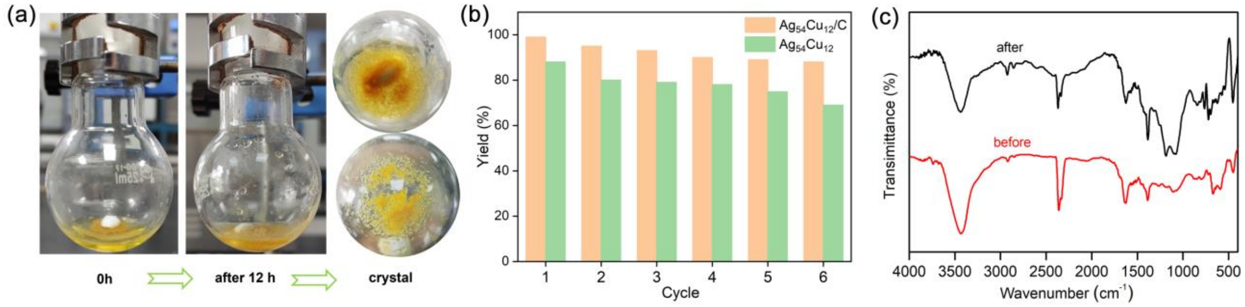


**Table S6.** The crystal data of production under **$Ag_{54}Cu_{12}$** as catalyst.

| Production | catalyst | Photograph of structure |
|---|---|---|
| 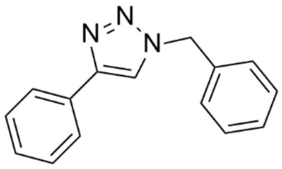 | **$Ag_{54}Cu_{12}$** | 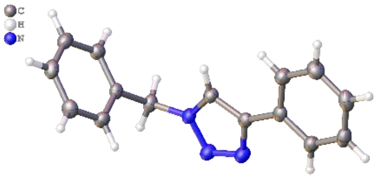 |
| 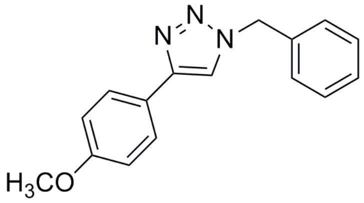 | **$Ag_{54}Cu_{12}$** | 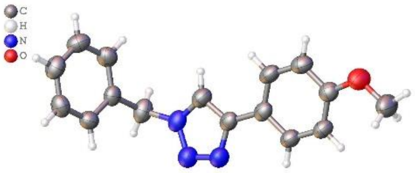 |
| 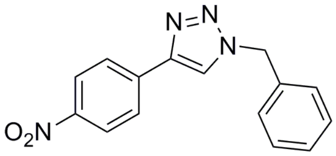 | **$Ag_{54}Cu_{12}$** | 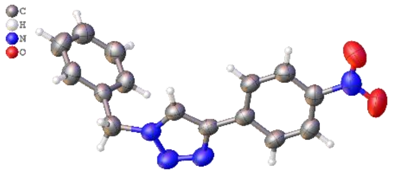 |

# NMR Spectra of the Products Derived from Cycloaddition of Alkyne and Azide

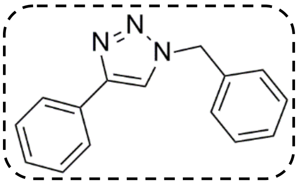

White crystal; $^1$H NMR (600 MHz, $CDCl_3$) δ 7.80 (d, *J* = 7.3 Hz, 1H), 7.66 (s, 0H), 7.43–7.36 (m, 2H), 7.32 (dd, *J* = 6.7, 4.0 Hz, 1H), 5.59 (s, 2H). $^{13}$C NMR (150 MHz, $CDCl_3$) δ 129.19, 128.82, 128.14, 125.71, 119.46, 54.26.

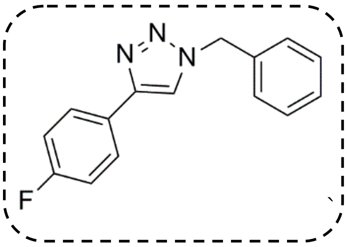

White solid; $^1$H NMR (600 MHz, $CDCl_3$) δ 7.77 (dd, J = 8.7, 5.4 Hz, 2H), 7.61 (s, 1H), 7.42–7.37 (m, 3H), 7.33–7.30 (m, 2H), 7.09 (t, J = 8.7 Hz, 2H), 5.57 (s, 2H). $^{13}$C NMR (150 MHz, $CDCl_3$) δ 162.46, 160.82, 146.37, 128.17, 127.83, 127.07, 126.41, 118.17, 114.84, 114.69, 53.26.

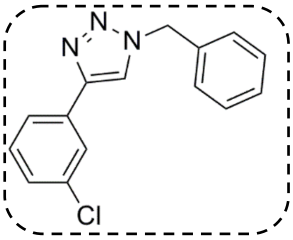

White solid; $^1$H NMR (600 MHz, $CDCl_3$) δ 7.73 (d, *J* = 11.8 Hz, 1H), 7.62 (d, *J* = 7.7 Hz, 1H), 7.59 (s, 1H), 7.33 (d, *J* = 7.2 Hz, 2H), 7.27–7.23 (m, 2H), 7.21 (d, *J* = 8.2 Hz, 2H), 5.51 (s, 2H). $^{13}$C NMR (150 MHz, $CDCl_3$) δ = 134.79, 134.46, 132.32, 130.11, 129.25, 128.94, 128.15, 125.78, 123.77, 119.81, 54.36.

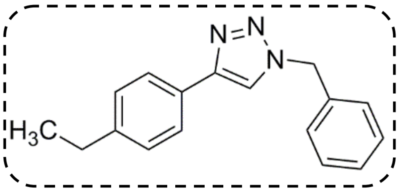

White solid; $^1$H NMR (600 MHz, $CDCl_3$) δ 7.71 (d, *J* = 7.7 Hz, 2H), 7.62 (s, 1H), 7.38 (d, *J* = 7.4 Hz, 3H), 7.31 (d, *J* = 6.5 Hz, 2H), 7.23 (d, *J* = 7.6 Hz, 3H), 5.57 (s, 2H), 2.66 (q, *J* = 7.4 Hz, 2H), 1.24 (t, *J* = 7.5 Hz, 4H). $^{13}$C NMR (150 MHz, $CDCl_3$) δ 134.78, 129.16, 128.77, 128.31, 128.06, 125.70, 119.14, 54.22.

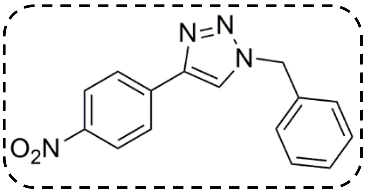

White solid; $^{1}$H NMR (600 MHz, $CDCl_3$) δ 8.27 (d, *J* = 8.8 Hz, 2H), 7.97 (d, *J* = 8.8 Hz, 2H), 7.79 (s, 1H), 7.42 (q, *J* = 6.1 Hz, 3H), 7.37–7.30 (m, 2H), 5.61 (s, 2H). $^{13}$C NMR (150 MHz, $CDCl_3$) δ 128.31, 128.08, 127.20, 125.12, 123.27, 119.87, 98.96, 53.48.

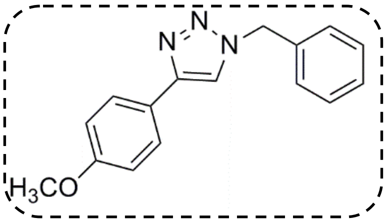


White solid; $^{1}$H NMR (600 MHz, $CDCl_3$) δ 7.72 (d, *J* = 8.7 Hz, 2H), 7.57 (s, 1H), 7.38 (t, *J* = 8.4 Hz, 3H), 7.31 (d, *J* = 6.7 Hz, 3H), 6.93 (d, *J* = 8.7 Hz, 2H), 5.57 (s, 2H), 3.83 (s, 3H). $^{13}$C NMR (150 MHz, $CDCl_3$) δ 128.12, 127.03, 125.98, 113.18.

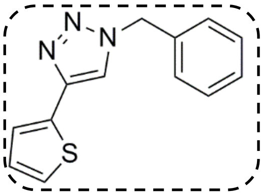


White solid; $^{1}$H NMR (600 MHz, $CDCl_3$) δ 7.57 (s, 0H), 7.39 (dt, *J* = 14.9, 5.2 Hz, 2H), 7.33 (dd, *J* = 3.5, 0.9 Hz, 1H), 7.32–7.29 (m, 1H), 7.27 (dd, *J* = 5.0, 0.9 Hz, 0H), 5.55 (s, 2H). $^{13}$C NMR (150 MHz, $CDCl_3$) δ 134.48, 132.85, 129.21, 128.89, 128.12, 127.61, 125.08, 124.19, 119.00, 54.30.

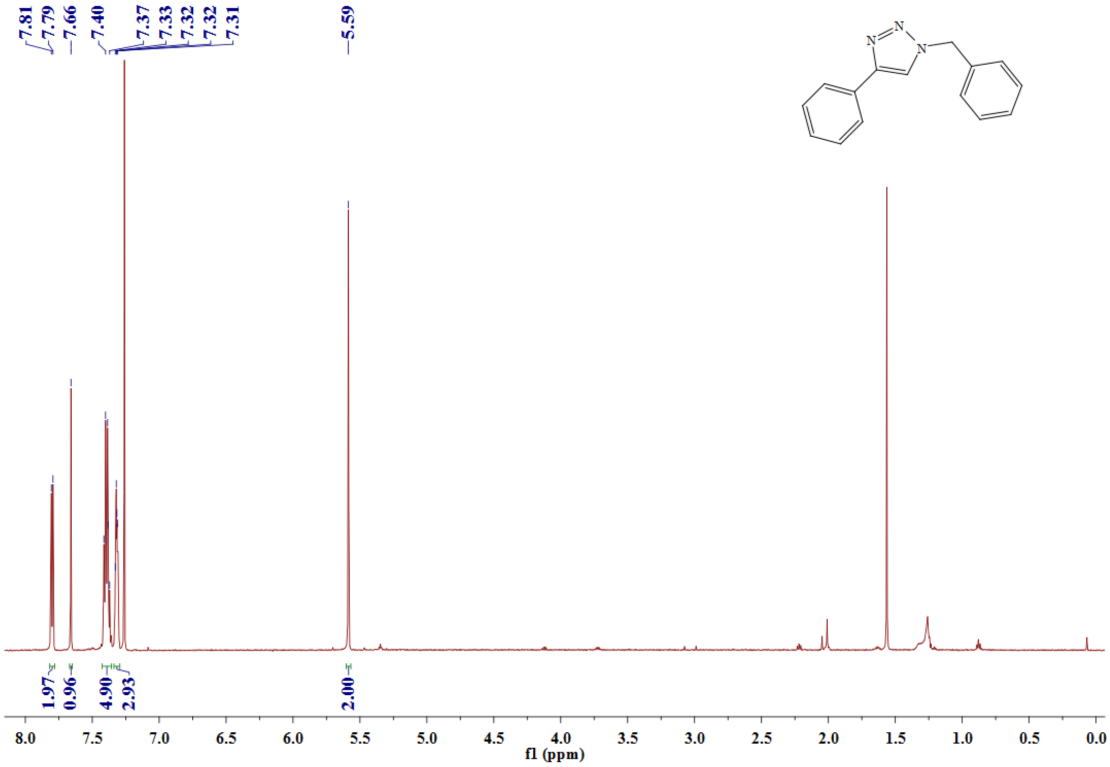
7.81
7.79
7.66
7.40
7.37
7.33
7.32
7.32
7.31
5.59
N
N
N
1.97
0.96
4.90
2.93
2.00
8.0 7.5 7.0 6.5 6.0 5.5 5.0 4.5 4.0 3.5 3.0 2.5 2.0 1.5 1.0 0.5 0.0
f1 (ppm)

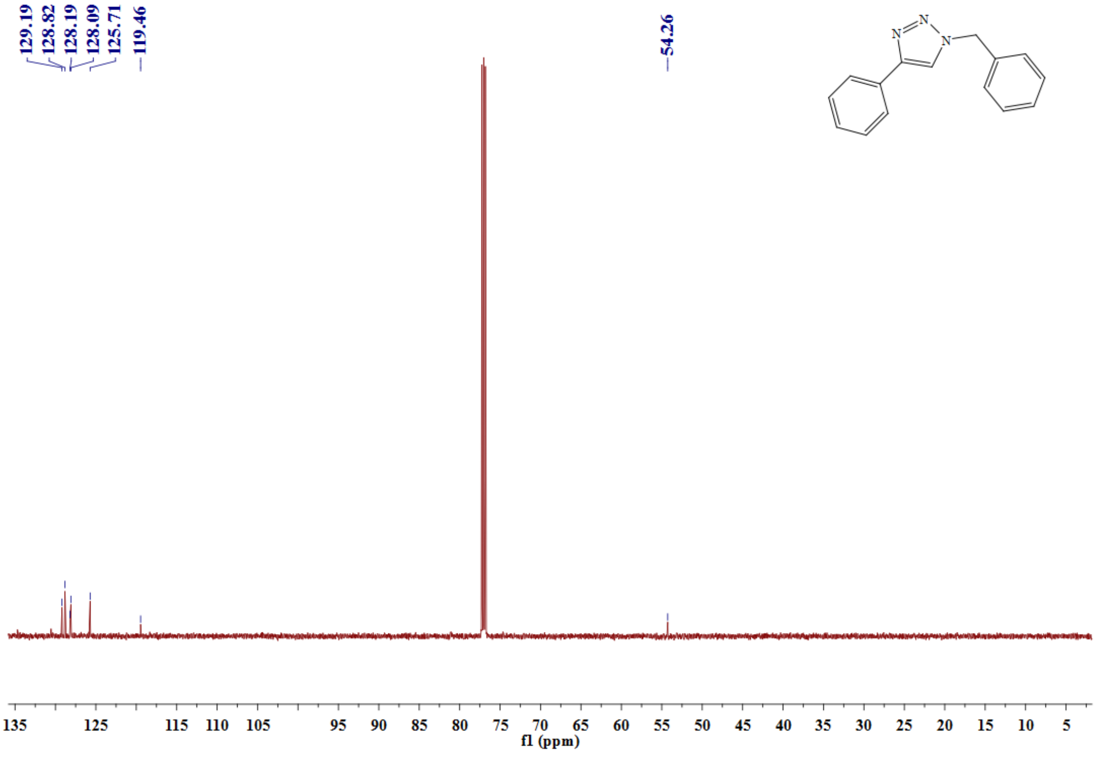
129.19
128.82
128.19
128.09
125.71
119.46
54.26
N
N
N
135 125 115 110 105 95 90 85 80 75 70 65 60 55 50 45 40 35 30 25 20 15 10 5
f1 (ppm)

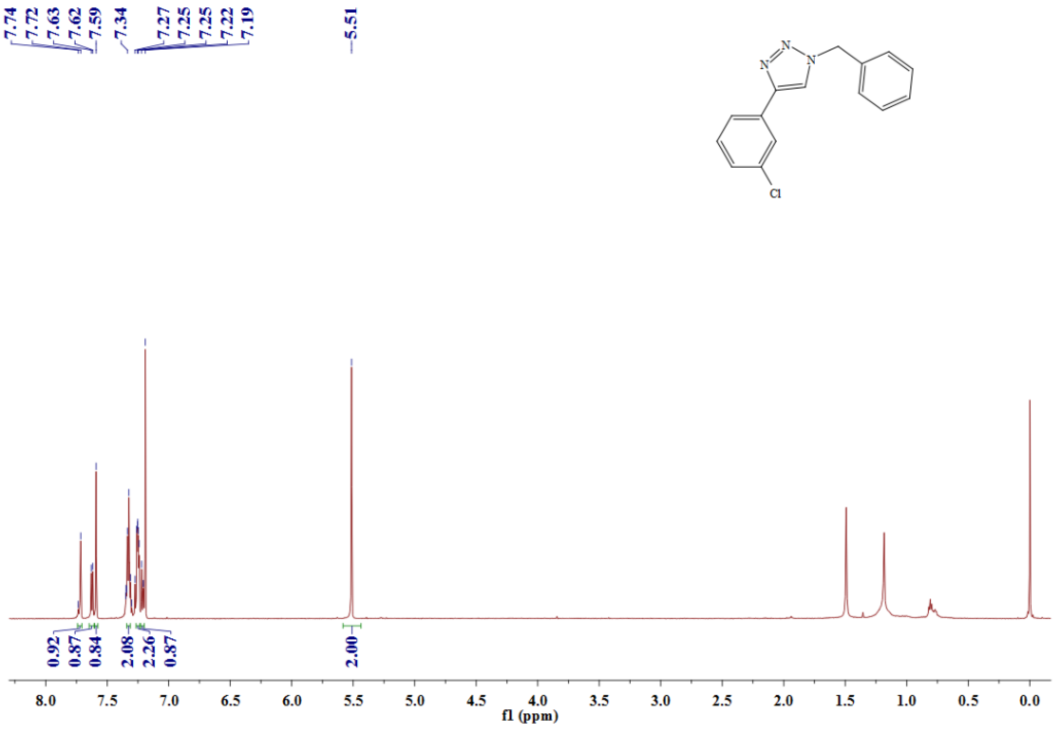
7.74
7.72
7.63
7.62
7.59
7.34
7.27
7.25
7.25
7.22
7.19
5.51
N
N
N
Cl
0.92
0.87
0.84
2.08
2.26
0.87
2.00
8.0 7.5 7.0 6.5 6.0 5.5 5.0 4.5 4.0 3.5 3.0 2.5 2.0 1.5 1.0 0.5 0.0
f1 (ppm)

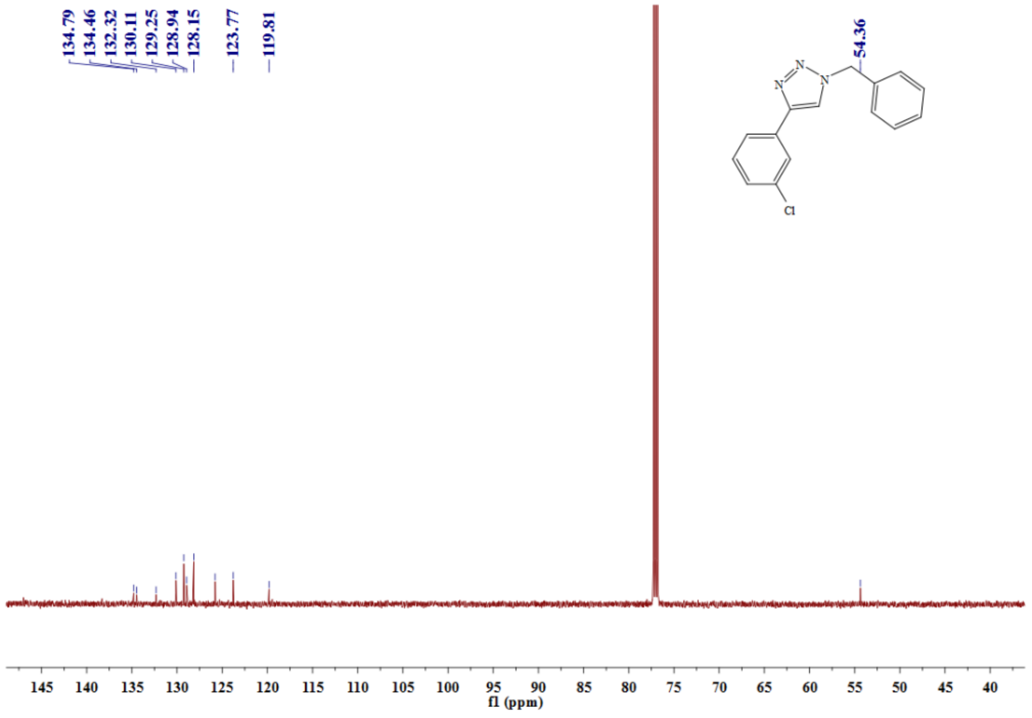
134.79
134.46
132.32
130.11
129.25
128.94
128.15
123.77
119.81
54.36
N
N
N
Cl
145 140 135 130 125 120 115 110 105 100 95 90 85 80 75 70 65 60 55 50 45 40
f1 (ppm)

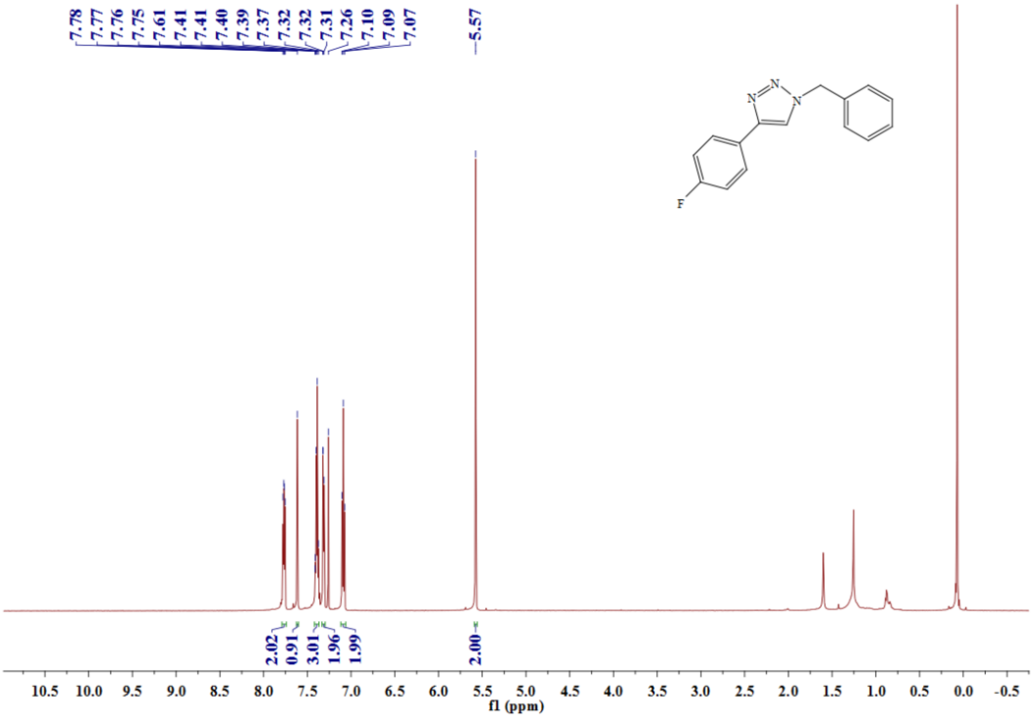
7.78
7.77
7.76
7.75
7.61
7.41
7.41
7.40
7.39
7.37
7.32
7.32
7.31
7.26
7.10
7.09
7.07
5.57
2.02
0.91
3.01
1.96
1.99
2.00
F
N
N
N
fl (ppm)

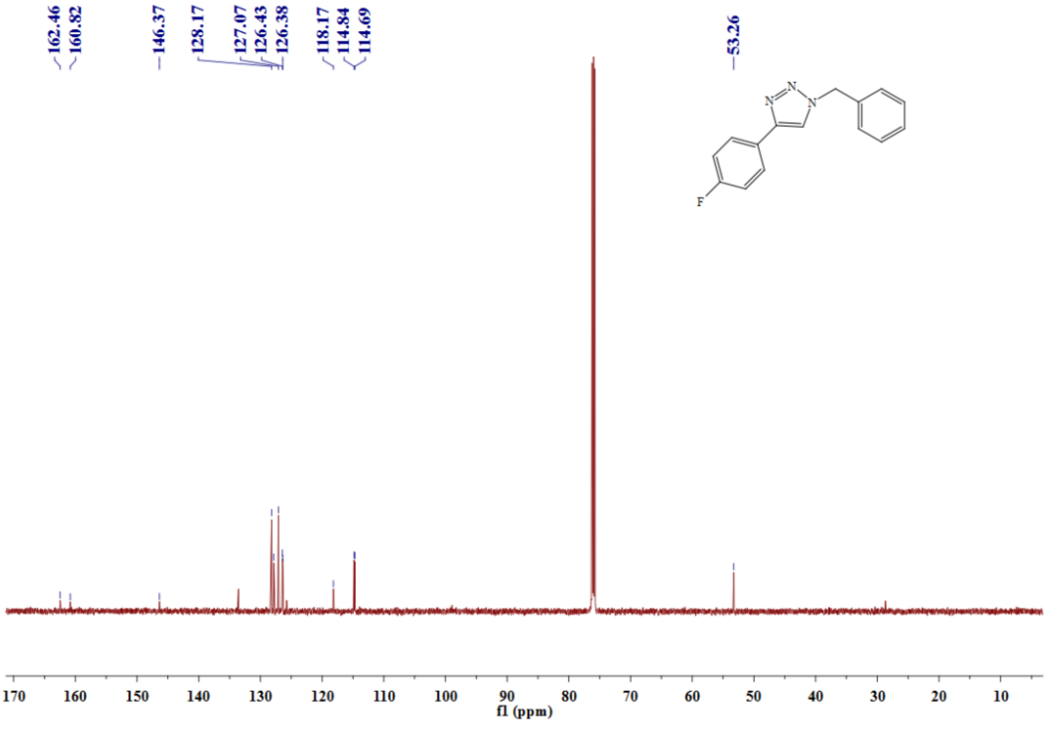
162.46
160.82
146.37
128.17
127.07
126.43
126.38
118.17
114.84
114.69
53.26
F
N
N
N
fl (ppm)

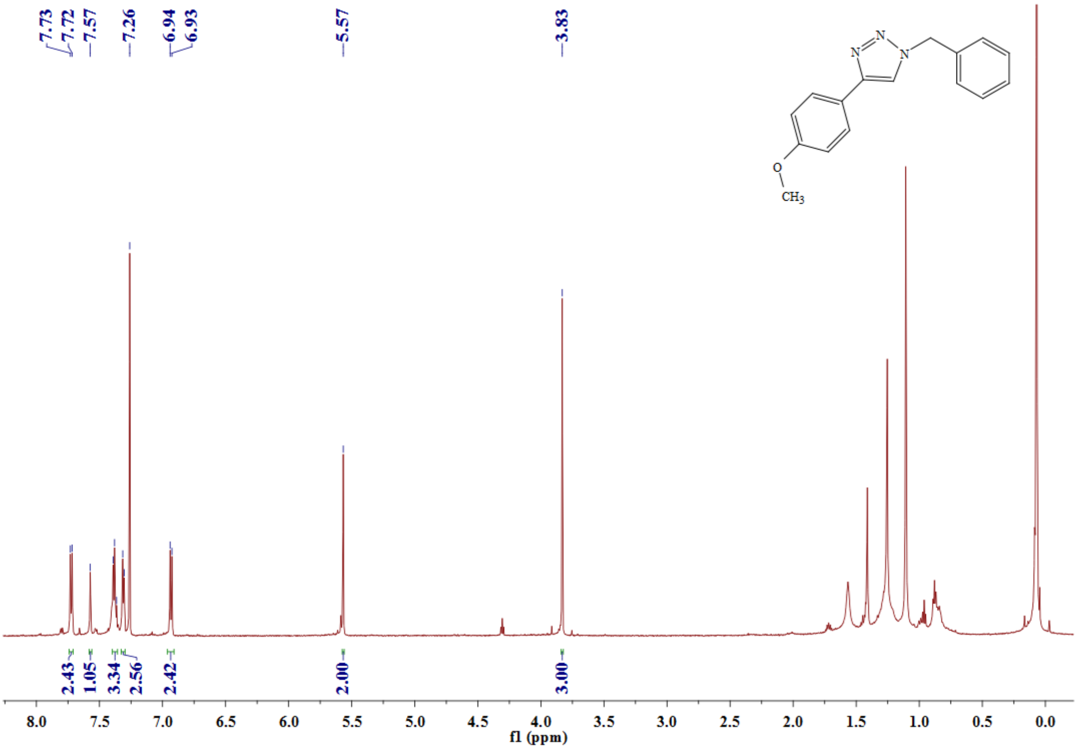
7.73
7.72
7.57
7.26
6.94
6.93
5.57
3.83
2.43
1.05
3.34
2.56
2.42
2.00
3.00
8.0 7.5 7.0 6.5 6.0 5.5 5.0 4.5 4.0 3.5 3.0 2.5 2.0 1.5 1.0 0.5 0.0
f1 (ppm)

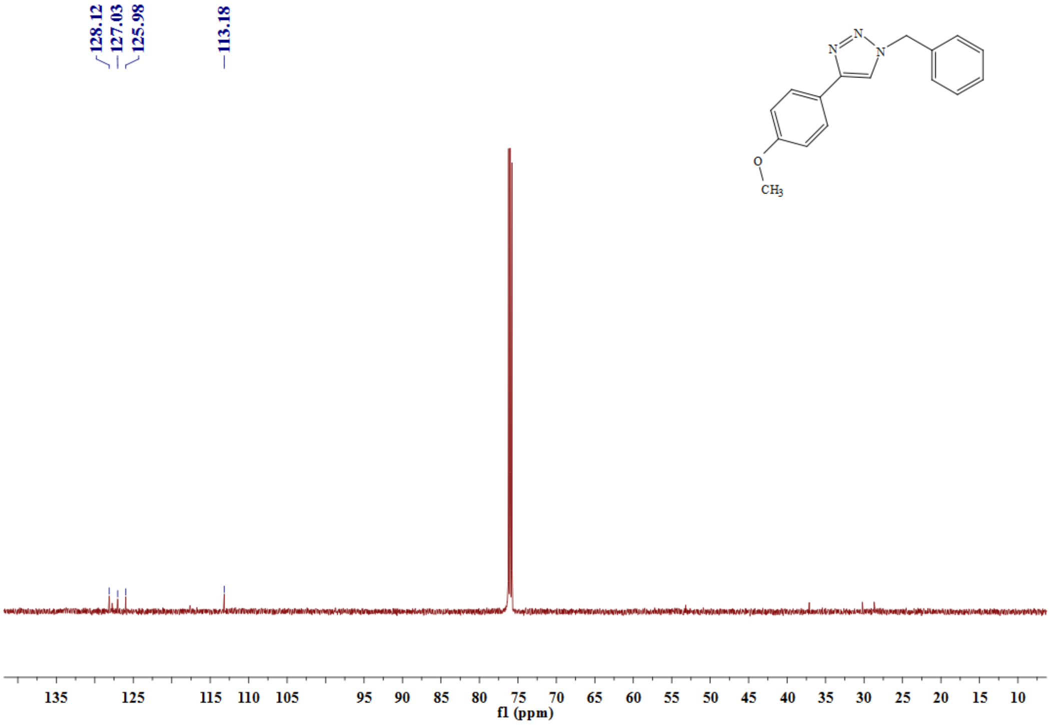
128.12
127.03
125.98
113.18
135 125 115 110 105 95 90 85 80 75 70 65 60 55 50 45 40 35 30 25 20 15 10
f1 (ppm)

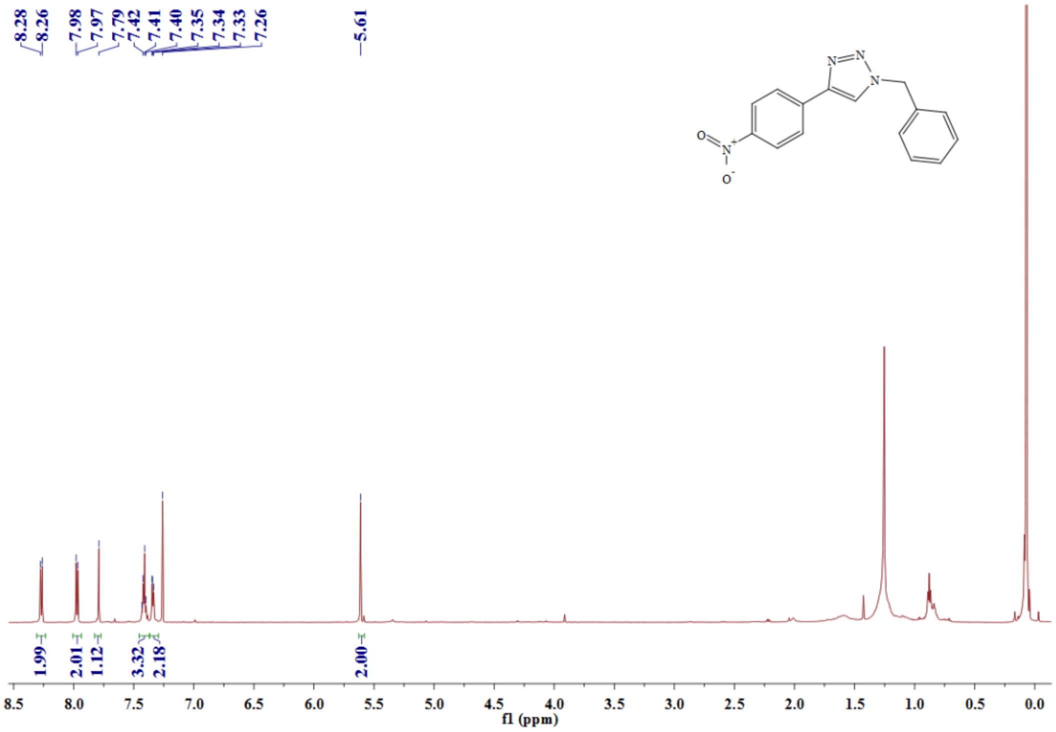
8.28
8.26
7.98
7.97
7.79
7.42
7.41
7.40
7.35
7.34
7.33
7.26
5.61
1.99
2.01
1.12
3.32
2.18
2.00
8.5 8.0 7.5 7.0 6.5 6.0 5.5 5.0 4.5 4.0 3.5 3.0 2.5 2.0 1.5 1.0 0.5 0.0
f1 (ppm)

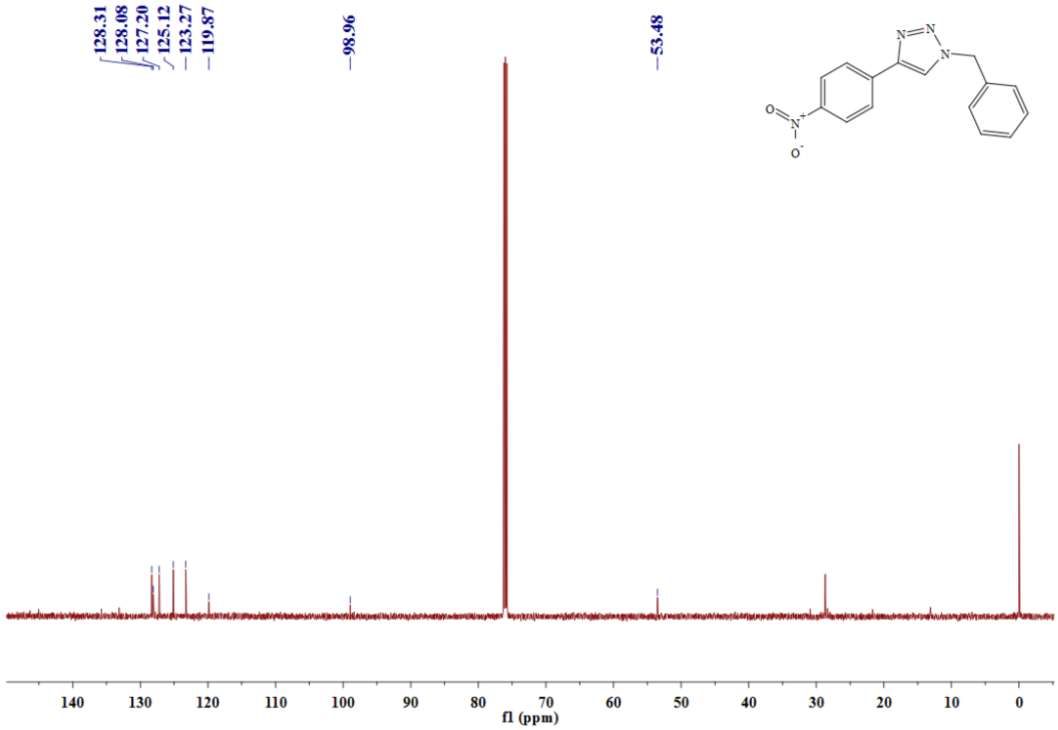
128.31
128.08
127.20
125.12
123.27
119.87
98.96
53.48
140 130 120 110 100 90 80 70 60 50 40 30 20 10 0
f1 (ppm)

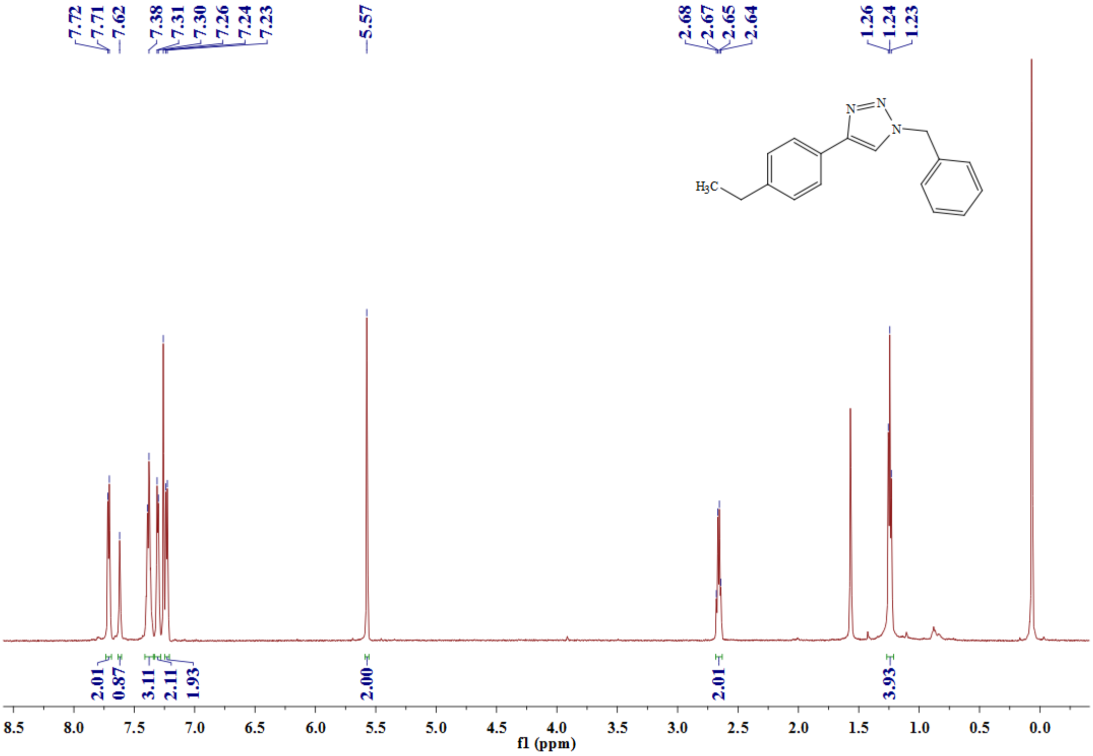
7.72
7.71
7.62
7.38
7.31
7.30
7.26
7.24
7.23
5.57
2.68
2.67
2.65
2.64
1.26
1.24
1.23
$H_3C$
N
N
N
2.01
0.87
3.11
2.11
1.93
2.00
2.01
3.93
8.5 8.0 7.5 7.0 6.5 6.0 5.5 5.0 4.5 4.0 3.5 3.0 2.5 2.0 1.5 1.0 0.5 0.0
f1 (ppm)

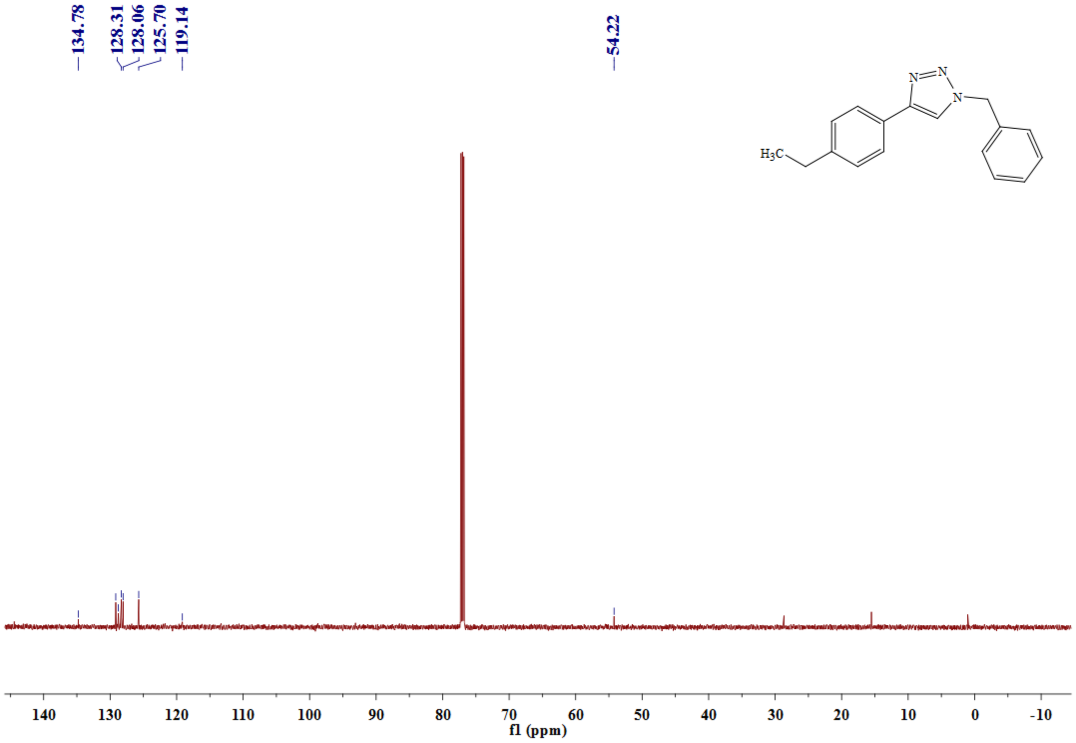
134.78
128.31
128.06
125.70
119.14
54.22
$H_3C$
N
N
N
140 130 120 110 100 90 80 70 60 50 40 30 20 10 0 -10
f1 (ppm)

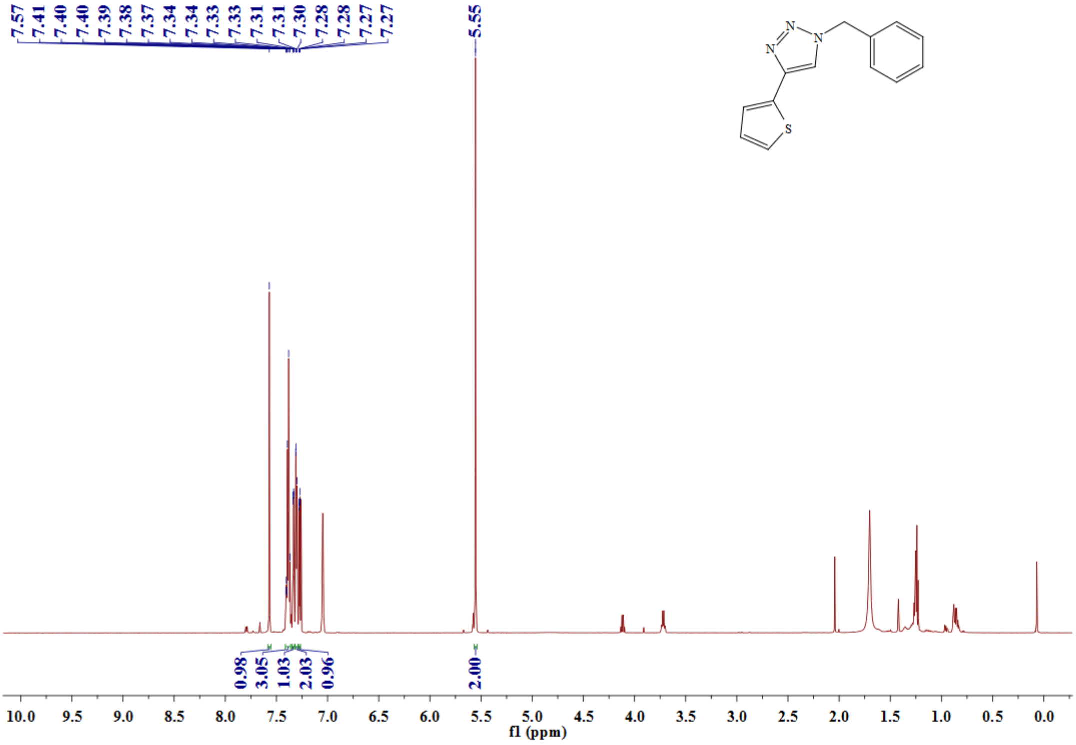

7.57
7.41
7.40
7.40
7.39
7.38
7.37
7.34
7.34
7.33
7.33
7.31
7.31
7.30
7.28
7.28
7.27
7.27
5.55
N
N
N
S
0.98
3.05
1.03
2.03
0.96
2.00
10.0 9.5 9.0 8.5 8.0 7.5 7.0 6.5 6.0 5.5 5.0 4.5 4.0 3.5 3.0 2.5 2.0 1.5 1.0 0.5 0.0
f1 (ppm)


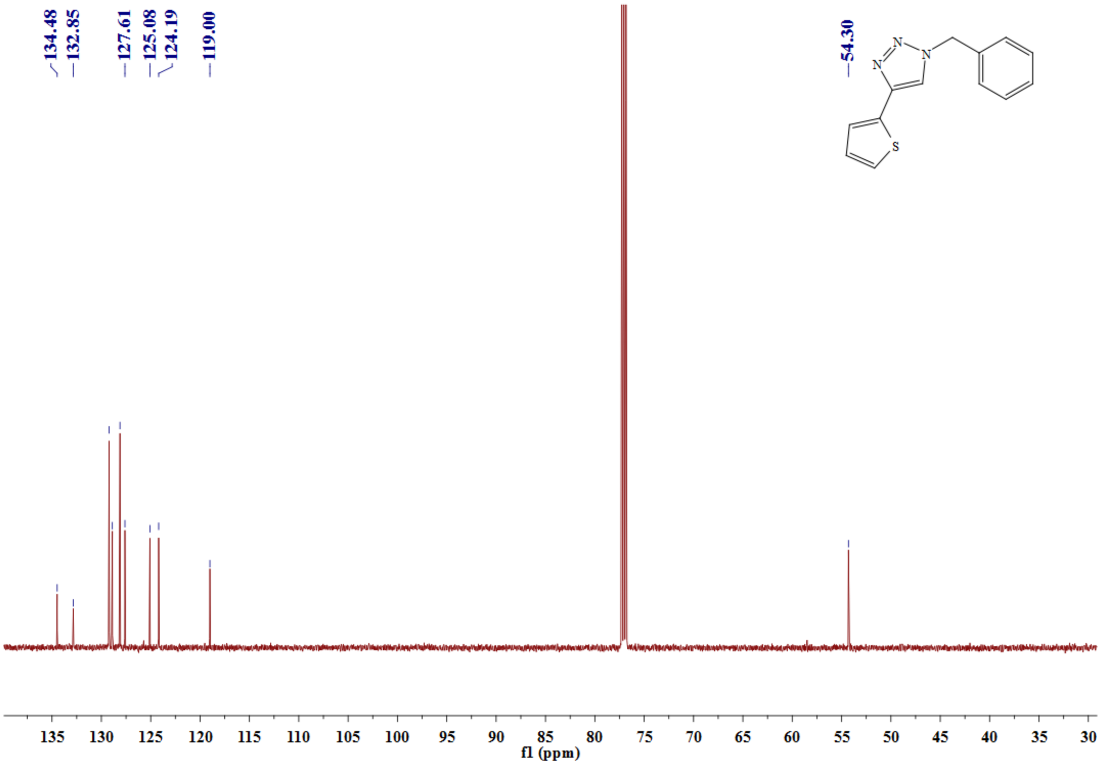

134.48
132.85
127.61
125.08
124.19
119.00
54.30
N
N
N
S
135 130 125 120 115 110 105 100 95 90 85 80 75 70 65 60 55 50 45 40 35 30
f1 (ppm)

## 4. Supporting references


S1. a) Rigaku Oxford Diffraction. CrysAlisPro Software system, version 1.171.40.68a, Rigaku Corporation: Oxford, UK, 2018; b) Rigaku Oxford Diffraction. CrysAlisPro Software system, version 171.40.19a, Rigaku Corporation: Oxford, UK, 2018.

S2. Palatinus, L.; Chapuis, G. J. Appl. Crystallogr. 2007, 40, 786. Dolomanov, O. V.; Bourhis, L. J.; Gildea, R. J.; Howard, J. A. K.; Puschmann, H. J Appl. Crystallogr. 2009, 42, 339.

S3. a) F. Hu, J.-J. Li, Z.-J. Guan, S.-F. Yuan, Q.-M. Wang, Angew. Chem. Inter. Ed. 2020, 59, 5312-5315; b) Z.-G. Jiang, K. Shi, Y.-M. Lin, Q.-M. Wang, Chem. Commun. 2014, 50, 2353-2355.
